# Family Caregivers' Experiences of Services for Children With Medical Complexity: A Systematic Review and Qualitative Evidence Synthesis

**DOI:** 10.1111/hex.70452

**Published:** 2025-09-29

**Authors:** Bethan Page, Pru Holder, Katie Doidge, Lisa Hinton, Lorna K. Fraser

**Affiliations:** ^1^ Cicely Saunders Institute of Palliative Care, Policy and Rehabilitation King's College London London UK; ^2^ Nuffield Department of Primary Care Health Science University of Oxford Oxford UK

**Keywords:** family caregivers, medical complexity, paediatrics, parents, qualitative health research

## Abstract

**Background:**

Many high‐income countries are seeking to adapt services to meet the needs of the growing population of children with medical complexity and their families but concerns have been raised about the quality of this care. To understand family caregivers' experiences of services and identify priorities for improvement we need to synthesise research about families' experiences of services for children with medical complexity.

**Objectives:**

To systematically identify and synthesise the qualitative evidence of family caregivers' experiences of health, care and education services for children with medical complexity.

**Methods:**

Systematic searches were conducted in MEDLINE, CINAHL, EMBASE, PsycINFO and ERIC from January 2011 to March 2024. Studies were assessed for methodological quality and data richness and synthesized using thematic synthesis.

**Results:**

Seventy‐one studies met the eligibility criteria. A purposive sample of 29 studies was taken, selecting good‐quality papers with rich data. These studies described the experience of 524 family caregivers and focused mostly on hospital care and care in the home. No studies were identified that focused specifically on family caregivers' experiences of education or social care services. Most studies were from the United States and Canada. The overarching theme was ‘concern for child's safety’ with three subthemes: ‘interactions with professionals’, ‘caring for the whole family’ and ‘system organisation’.

**Conclusions:**

Family caregivers' priority is maintaining their child's safety across all settings of care. Fragmented systems and difficulties trusting professionals exacerbate parents' stress and concern for their child's safety. To keep the child safe and well, services need to address the needs of the whole family (e.g., parental sleep and mental health, finances, housing). Future research is needed to address the gap in research on social care services and education.

**Patient and Public Contribution:**

Emerging findings of the review were discussed in a 2‐h workshop with six parents of children with medical complexity. The parents inputted into the development of the analytical themes and helped to shape the findings of the review.

## 1 Introduction

Advances in medicine have led to a growing population of children with medical complexity [1]. Children with medical complexity are defined as having (i) substantial family identified needs, (ii) at least one chronic condition which is severe and/or associated with medical fragility, (iii) severe functional limitations often associated with technology dependence (e.g., tube fed, long‐term ventilation) and (iv) high health care usage with ongoing involvement of multiple service providers [2, 3]. Many different services across health, social care, education and third‐sector organisations are involved in the care of these children. Concerns have been raised that services are not meeting the needs of these children, with families reporting difficulties navigating the system [4, 5, 6, 7], problems with the safety of care [8, 9, 10] and issues accessing the required equipment, housing and services [5, 10, 11]. Parents have described their stress as a direct result of their battles with services rather than as a result of caregiving [12]. Health, social care and education systems need to adapt to meet the needs of the growing population of children with medical complexity.

Internationally, services are seeking to make changes to care models, but there is a lack of evidence on how best to do this [13, 14, 15, 16]. Understanding the experiences and perspectives of parents on the services supporting children with medical complexity will help to inform improvements to services. There have been a number of primary qualitative studies published that report on parents and family caregivers' experiences, but there is no up‐to‐date review that draws the evidence together on parents' experiences of care across countries and settings for children with medical complexity.

This aim of this review is therefore to systematically identify and conduct a qualitative evidence synthesis of the existing research to understand family caregivers' experiences of health, care and education services for children with medical complexity.

## 2 Methods

This review was registered with PROSPERO (CRD42024526885) and reported in accordance with Enhancing Transparency in Reporting the synthesis of Qualitative research (ENTREQ) guidelines [17].

### 2.1 Search Strategy

A comprehensive search strategy was developed using subject terms and synonyms for the following search concepts: (i) ‘medical complexity’, (ii) ‘parent/caregiver’ (iii) ‘experiences’ (File S1). A search filter was included in the search to identify qualitative research. We use the term caregiver to include family caregivers such as grandparents or older siblings who have custody of children and are the child's primary caregiver. Five bibliographic databases were searched (MEDLINE, EMBASE, CINAHL, PsycINFO and ERIC) from January 2011, which is when Cohen's seminal paper on the definition of medical complexity was published. The medical complexity of children has increased due to medical advancements and increased life expectancy, so studies from before this date were deemed to be less relevant to current practice. Furthermore, before 2011, the term ‘medical complexity’ did not have a clear definition and was not an established term in the literature. Forward citation searching was also conducted on Cohen et al.'s paper (a seminal paper in the field) to check for any additional papers [2].

Title and abstract screening was conducted by one author with a sample of 20% checked by a second author. The full texts of potentially relevant studies were screened independently by two authors (BP & PH), with any disagreements resolved through discussion with a third reviewer (L.F.). The screening was completed through Covidence with regular team meeting to discuss any conflicts.

### 2.2 Eligibility Criteria

The full list of inclusion and exclusion criteria is given in Table 1.

**Table 1:** Inclusion and exclusion criteria.

	Included	Excluded
Study type	Primary qualitative studies and mixed method studies with a separately reported qualitative analysis, and qualitative data from questionnaire surveys if analysed separately Studies published in English language, in peer reviewed journals after 1 January 2011, until March 2024. This was the year Cohen et al., published their paper defining the population of children with medical complexity. The review will be restricted to understanding relatively recent experiences of services, as old service models before this time period may be outdated now, and complexity of children's needs have increased in recent years due to improvements in medical treatments.	Studies that are not qualitative studies, or do not include a qualitative analysis: quantitative studies, systematic reviews, comments, letters, editorials, notes, news, newspaper articles, protocols, conference abstracts, literature reviews. Studies in which qualitative data has been analysed quantitatively. Studies not published in English.
Population	Studies reporting the experiences of family caregivers of children with medical complexity. We use the term family caregiver to encompass mothers, fathers, step‐parents, adoptive parents, legal guardians and long‐term foster parents, recognising that some legal guardians may be other relatives such as grandparents. We also include bereaved parents. Studies with mixed samples that include the views of other relatives such as grandparents or siblings or of children themselves, if > 70% are parents, or there is a separate analysis of parent data. This decision was to ensure a focus on primary caregivers, the majority of whom are parents. Studies with mixed samples that include healthcare professionals if there is a separate analysis of family caregiver data.	Studies which include children with behavioural conditions such as ADHD and autism in the absence of clear evidence in the paper of meeting all four domains of Cohen's criteria, including functional limitations that are severe and may require assistance from medical technologies. If children had behavioural issues alone, the study would not be included. Studies which report on the experiences of health care professionals without reporting separately the experiences of parents. Studies that are reporting only the experience of grandparents, siblings, or other relatives of children with medical complexity. Studies where none of the results/findings are relevant to experiences of services or what parents need from services. Studies which focus on adults > 18 years, or specifically on issues of transition to adult services.

### 2.3 Data Extraction

All eligible studies were identified and information such as author, year of publication, country, setting, aims, methodology, family caregiver participation, age of children, conditions of children and findings were extracted and organised into a data extraction table. Data extraction was completed by one author (K.D.), and checked by two others (B.P. and P.H.).

### 2.4 Purposive Sampling

In qualitative evidence synthesis, including too many papers in your review can threaten the quality of the synthesis: the more data there is to synthesise, the less depth and richness the researchers are able to extract from the data [18]. Due to the large number of relevant studies identified, a two‐stage sampling framework was used to purposively select studies to be included in the review and take forward for thematic synthesis by sampling for data richness and maximum variation [18]. First, studies were scored on a scale of one to five based on data richness in relation to the research question for this review [18]: the scoring was carried out by two authors independently. Any conflicts were discussed by the two authors and resolved through discussion with a third author. In the second stage, the studies were mapped by country and setting of care to ensure maximum variation so that no relevant data was missed. All studies which scored 4 or 5 were included. Most of these studies were from Canada or the United States. Our focus in this review was to inform service improvement within the UK context: due to limited research on children with medical complexity being conducted in the United Kingdom, the authors included all UK‐based studies if they scored 3 or higher, which resulted in an additional two included studies. This sample captured a broad range of study settings.

### 2.5 Quality Appraisal

The Critical Appraisal Skills Programme (CASP) tool [19] was used to conduct quality assessment of the studies included in the thematic synthesis. This was conducted by one reviewer and checked by two authors. Following the quality assessment, a further two studies which were rated as ‘some concerns’ were removed from the sample for thematic synthesis.

### 2.6 Data Synthesis

Three inductive stages described by Thomas and Harden were used to conduct the thematic synthesis [20]. This established method enabled the researchers to aggregate and interpret existing qualitative evidence clearly and transparently.

In stage 1, each line of text from the result section of the included studies was line‐by‐line coded according to its meaning and content using NVivo. New codes were added inductively, and each sentence was given at least one code until a comprehensive bank of codes were developed. The first stage was carried out by one reviewer, with regular input from the review team on codes and concepts. The review team completed Stages 2 (developing descriptive themes) and 3 (generating analytical themes) together, meeting regularly to discuss and develop the analytical themes. The grouping of the codes was also discussed in a 2‐h workshop with six parents who were part of an existing parent advisory group for research on children with life‐limiting conditions and medical complexity. The parents were presented with all the codes emerging from the data and illustrative quotes, and discussed their thoughts on the developing analytical themes. The group included both bereaved and non‐bereaved parents of children with medical complexity of a range of ages. Five were mothers and one a father. In the write up, each subtheme is illustrated with direct participant quotations.

### 2.7 Reflexivity

The members of the review team had a diverse background and met regularly throughout the review process. Two authors were health services researchers with backgrounds in psychology (B.P. and P.H.), one a paediatric clinical academic (L.F.), a medical student (K.D.) and a qualitative methods expert (L.H.).

## 3 Results

**Figure 1:**
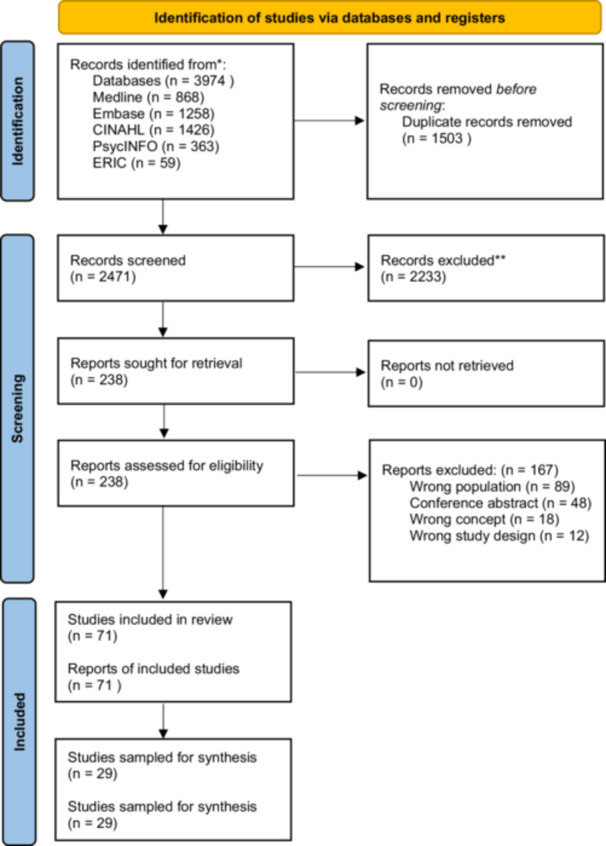
Preferred Reporting Items for Systematic Reviews (PRISMA) flowchart showing the inclusion of 29 studies from the 3974 identified.

3974 records were identified from the databases and 238 full texts were screened against the eligibility criteria (Figure 1). From the eligible 71 studies, 29 were selected for the synthesis following purposive sampling. File S2 gives details of the 42 reports that met the search criteria but were not included in the synthesis following sampling.

### 3.1 Study Characteristics

The characteristics of the included studies are shown in Table 2. The studies included in the synthesis were undertaken in five high‐income countries: the United States (*n *= 13), Canada (*n *= 8), United Kingdom (*n *= 5), Australia (*n *= 2) and Sweden (*n *= 1). They were published from 2011 to 2024, with 20 published since 2020. Four studies focused on experiences of services during or shortly after the COVID‐19 Pandemic.

**Table 2:** Summary of included studies.

Authors	Year of publication	Country	Setting	Aims	Methodology	Condition of children	Age of children	Parent participants	Analysis methodology	Summary of methodological assessment
Amar‐Dolan et al.	2020	USA	Transition from hospital to home	This study explores the experience of family caregivers of children and young adults with a tracheostomy during the transition from hospital to home care. We sought to identify the specific unmet needs of families to direct future interventions.	Semistructured interviews, focus groups	Mixed conditions	Range = 9 month–28 years	Mothers, *n* = 10 fathers, *n* = 1 young adult, *n* = 1	Grounded‐theory approach	No concerns
Ames et al.	2023	USA	All health and care settings	To qualitatively describe the perspectives and experiences of family caregivers of children with medical complexity regarding disability‐based discrimination in health care.	Semistructured interviews	Not reported	Range = 18 months–18 years	30 participants female, *n* = 25 (83.3%)	Thematic analysis	No major concerns
Ames et al.	2024	USA	All health and care settings	To qualitatively assess the impact of disability‐based discrimination in healthcare on the parents of children with medical complexity (CMC).	In‐depth, semistructured interviews	Not reported	Range = 6 months–5 years	30 parents female, *n* = 25 (83.3%)	Thematic analysis	No major concerns
Boss et al.	2020	USA	Home	To explore the lived experiences of a nationwide sample of families of children who have received paediatric home healthcare.	Telephone interviews, semi‐structured surveys	Not reported	Not recorded	48 parents (2 from same family)	Conventional content analysis	No major concerns
Buchanan et al.	2022	Canada	All health and care settings (recruited via hospital)	To address the gaps in the current approach to shared decision‐making for children with medical complexity (CMC) by better understanding the decision‐making activity among parents of CMCs and exploring what comprises their decision‐making activity.	Semistructured interviews	Neurological disorders including cerebral palsy; rare diseases; and complex respiratory issues, including those requiring a tracheostomy and mechanical ventilation.	Not recorded	12 participants Mothers, *n* = 10 father, *n* = 1 young adult, *n* = 1 (formerly [child] with medical complexity)	Activity theory: familiarization, thematic analysis, indexing, charting, and mapping and interpretation	No major concerns
Cady et al.	2017	USA	Care coordination service	To understand how the parents of medically complex children enrolled in PRoSPer (primary‐speciality care coordination partnership for children with medical complexity) perceive communication and collaboration between the providers and services delivering care for their child.	Focus groups	Mixed conditions	Range = 0–11 years old	8 parents mothers, *n* = 5 fathers, *n* = 2	Conventional content analysis	No major concerns
Currie et al.	2023	Canada	Care coordination service	To examine the implications of pandemic restrictions on care coordination from caregiver perspectives.	Qualitative description, semistructured interviews	ASD and ADHD with medical complexity	Range 6–19 years	19 caregivers, mothers *n* = 13 fathers *n* = 4 grandmothers *n* = 2	Thematic analysis	No major concerns
Dewan et al.	2023	Canada	All health and care settings	To describe the experiences and impact of paediatric medical stress among parents of children with medical complexity	Semistructured interviews	Not reported	Range = ≤ 23 months–≥ 14 years	Mothers, *n* = 20 fathers, *n* = 2	Thematic analysis	No major concerns
Fong et al.	2024	Canada	Virtual care/telemedicine	To explore parent perspectives and experiences regarding their experience of virtual care during the pandemic.	Semistructured interviews	Not reported	Range = 1–≥ 19 years	Mothers, *n* = 28 fathers, *n* = 2	Theoretical coding	No major concerns
Fong et al.	2023	Canada	All health and care settings	To explore parents' perspectives on improving healthcare services and supports to better meet the needs of children with medical complexity and their families.	Semistructured interviews	Not reported	Range = 1–≥ 19 years	Mothers, *n* = 14 fathers, *n* = 2	Interpretive description	No major concerns
Foster et al.	2022	USA	Home	(1) To characterize which current supports parents use to care for CMC at home and any related gaps in that support, and (2) summarize parents' preferences for additional supports to minimize identified gaps.	Semistructured interviews	Mixed conditions	Range = 2–18 years	Mothers, *n* = 16 fathers, *n* = 2	Inductive analysis	No major concerns
Frush et al.	2023	USA	Virtual care/telemedicine	To describe the experiences of caregivers of CMC with postdischarge telemedicine video visits.	Semistructured telephone interviews	Not reported	Mean = 8 years	12 adult caregivers 11 were parents	Colaizzi's decriptive phenomenological method for thematic construction	No major concerns
Golden et al.	2012	USA	All health and care settings	To determine which aspects of care coordination are important to caregivers and explore the challenges faced by caregivers in coordinating care.	Semistructured disscusion	Not reported	Not recorded	Mothers, *n* = 11 fathers, *n* = 2 sister, *n* = 1	Thematic analysis	No major concerns
Hagvall et al.	2016	Sweden	Hospital	To describe parental experiences of caring for their child with medical complexity during hospitalization for acute deterioration, specifically focussing on parental needs and their experiences of the attitudes of staff.	Descriptive design and semistructured interviews	Gave examples of neurological conditions and autism	Range = 2–13 years	Mothers, *n* = 7 fathers, *n* = 2	Qualitative content analysis	No concerns
Hlyva et al.,	2021	Canada	Care coordination service/specialist clinic	This study explored parent engagement within the context of a feasibility study evaluating an Integrated Tertiary Complex Care (ITCC) clinic created to support children with medical complexity closer to home. This paper aimed: (1) to understand the family experiences of care and (2) to explore parent engagement in the study.	Focus groups, one‐on‐one interviews, questionnaires	Not reported	Not mentioned	Focus groups: 24 parents 19 (female *n* = 14, male *n* = 5) provided comments. One‐on‐one interviews: 17 participants (female *n* = 13, male *n* = 4) Questionnaire: 21 participants	Interim analysis, analysis using NVivo	No major concerns
Hobson et al.	2011	UK	All health and care services	To describe the experiences of fathers who cared for their children with complex health and nursing care needs. In particular, we wanted to understand better how fathers were managing their children's complex care and the meaning they attached to their roles.	In‐depth qualitative interviews	Epilepsy mentioned	Range =16 months–16 years	Fathers, *n* = 8	Analysed using Burnard's approach, which has commonalities with phenomenological and content analysis	No major concerns
Keilty et al.	2018	Canada	Home/respite	To explore experiences of parents of children with chronic respiratory conditions who have used unregulated respite to provide complex care at home for their child with a chronic respiratory condition.	Semistructured interviews	Not reported	Range = 1–18 years	Mothers, *n* = 20	Comparative analysis	No concerns
Leary et al.	2020	USA	Hospital	To elicit parent perspectives during hospital readmissions regarding the care of children with medical complexity (CMC) with the goal of identifying opportunistic areas to target interventions to improve care and experiences while potentially decreasing readmissions for CMC and their families.	Semistructured interviews	Mixed conditions	Range = 0–15	20 participants	Uploaded to Dedoose software for analysis by using a modified grounded theory approach, then analysed thematically	No major concerns
McLorie et al.	2023	UK	All health and care services (recruited via tertiary children's hospitals)	To understand parents' instances of receiving care for their child with medical complexities across England.	Semistructured interviews	Neurological and congenital/genetic conditions	Range = 0–20 years	18 interviews parents, *n* = 20 mothers, *n* = 14 fathers, *n* = 6	Reflective thematic analysis	No major concerns
Mendes et al.	2013	USA	Home (home nursing)	To elicit parents' views of ideal home nursing care for technology‐dependent children and supportive nursing interventions.	Semistructured interviews	Neurological, behavioural and sensory conditions	Range = 2.5–15 years	Participants, *n* = 7 couples, *n* = 3 mothers, *n* = 1	Qualitative content analysis, cross‐case analysis	No concerns
Mitchell et al.	2022	UK	Home	To identify the types of home adaptations that families require to care for their child at home; to explore family members' experiences of having these adaptations made to their home; and explore family members' satisfaction with these home adaptations.	Semistructured interviews, photo‐elicitation interviews	Not reported	Range = 5–25 years	12 parents mothers, *n* = 10 fathers, *n* = 2	Braun and Clarke's seven‐stage thematic analysis	No major concerns
Moyes et al.	2022	Australia	All health and care services	The aim of this study was to explore the support needs of parents who have a child with medical complexity living in the family home.	Semistructured interviews	Mixed conditions	Range = 2–16 years	Mothers, *n* = 12	Thematic analysis	No major concerns
Nageswaran et al.	2022	USA	All health and care services	To describe the types of communication challenges faced by spanish‐speaking parents with limited english proficiency, factors associated with these challenges, and their consequences on the healthcare received by CMC.	In‐person interviews, abstracted logs of care coordination tasks	Congenital/genetic, neurological conditions and prematurity	Range = 6 months–18 years	70 caregivers	Thematic content analysis, inductive analysis	No major concerns
Page et al.	2020	UK	All health and care services	To explore the challenges experienced by families caring for children who need complex medical care at home.	Unstructured followed by semistructured interviews	Mixed conditions	Range = 18 weeks–9 years	11 interviews mothers, *n* = 11 fathers, *n* = 4 couples, *n* = 4	Thematic analysis, supra analysis	No major concerns
Rennick et al.	2019	Canada	Paediatric Intensive Care	To explore the experiences of parents of children with medical complexity during paediatric intensive care (PICU) admission.	Semistructured interviews	Mixed conditions	Range = 10 months–18 years	Parents, *n* = 17 mothers, *n* = 10 (11 interviewed individually, 3 with spouses)	Constant comparative method	No major concerns
Sherman et al.	2024	USA	All health and care services	(1) to investigate the nature and dynamics of interactions within the mesosystem that affect primary caregivers of children with tracheostomies, (2) to explore the impact of mesosystem interactions on the well‐being and caregiving experiences of primary caregivers, and (3) to examine the connection between mesosystem interactions and broader healthcare inequities in the context of tracheostomy care in the United States.	Semistructured interviews	Not reported	Range = 16 months–11 years	Participants, *n* = 11 Mothers, *n* = 9	Analytical framework	No concerns
Thomas et al.	2012	UK/Wales	Home/respite	To explore parents' experience of caring for a child with complex health needs and to evaluate whether the nursing respite service at home meets their needs.	Semistructured, in‐depth interviews	Mixed conditions	Range = 3–14 years	Mothers, *n* = 7	Not mentioned	No major concerns
Welsh et al.	2014	Australia	Home/respite	The aim of the project was to determine what type of respite would be beneficial to the carers of children with complex health needs (CWCHN) and what barriers they face when trying to access respite.	Qualitative descriptive approach, semistructured interviews	Not reported	Not reported	Participants, *n* = 5	Thematic analysis	No major concerns
Yu et al.	2022	USA	Complex care service at hospital	To better understand CMC caregivers' perceptions of what constitutes high‐quality care at a complex care center.	In‐depth, semistructured interview	Mixed conditions	Range = 0–≥ 12 years	20 participants Biological parents, *n* = 18 (90%) Female, *n *= 19 (95%)	Thematic analysis	Some concerns

Studies focused on experiences of services across a variety of settings: general health and care services (*n *= 12), care at home (*n *= 4), respite provision (*n *= 3), care coordination services (*n *= 3) and virtual care/telemedicine (*n *= 2). Five studies focused specifically on hospital care, including general hospital services (*n *= 2), paediatric intensive care (*n *= 1), a complex care hospital service (*n *= 1) and the transition from hospital to home (*n *= 1).

The data collection method most frequently used was interviews (*n *= 27), followed by focus groups (*n *= 3) and surveys/questionnaires (*n *= 2). One study asked parents to create logs of their care coordination tasks. Four studies used a combination of methods. A variety of qualitative method were used across the studies. Thematic analysis was the most common analytical approach (*n *= 14), others included grounded theory and content analysis.

#### 3.1.1 Characteristics of Participants and Their Children

524 caregivers participated across the 29 studies. Some studies reported the exact relationship to the child, whereas others used general terms such as ‘caregiver’ or indicated whether the participant was male or female. Of the studies which did clearly report whether participants were mothers or fathers, there were 228 mothers and 40 fathers. Most of the studies reported the ages of the children, which ranged from 0 to 28 years old, with the majority under 19 years.

#### 3.1.2 Quality Appraisal of Included Studies

Table 2 includes the CASP appraisals of the purposively included studies: four had no concerns [5, 21, 22, 23], one had some concerns [24] and the remaining 23 had no major concerns. Full details are given in File S3.

### 3.2 Findings

#### 3.2.1 Themes: Concern for Child's Safety

**Figure 2:**
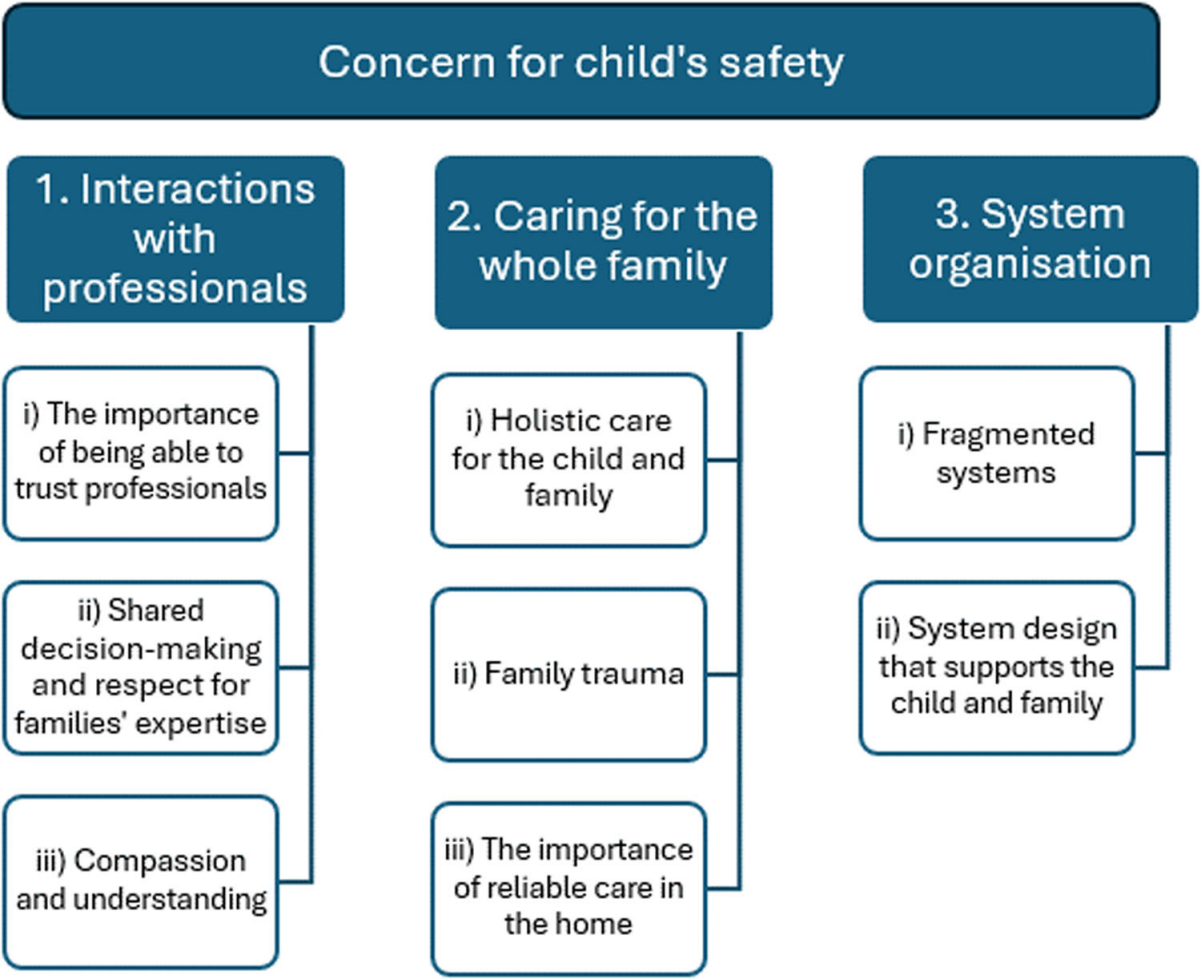
Thematic map.

The overarching theme was ‘concern for their child's safety’. Subthemes included: (i) ‘interactions with professionals’, (ii) ‘caring for the whole family’ and (iii) ‘system organisation’ (Figure 2). Concerns for the child's safety arose from negative interactions with professionals (e.g., a lack of trust in some professionals), insufficient family support and fragmented systems. Family caregivers wanted services to prioritise holistic family care, and above all to support them to keep their child safe.

#### 3.2.2 Interactions With Professionals

##### 3.2.2.1 The Importance of Being Able to Trust Professionals

Family caregivers emphasised the importance of being able to trust the professionals (healthcare professionals, paid carers etc.) caring for their child to carry out routine care and to manage emergencies. Instances of unsafe care, errors, or concerns about staff competency were described across hospital and paid care in the home [4, 5, 25].

We had an emergency where [child] was still very limp and not awake. The home nurse's ﬁrst instinct was to rub his sternum. She didn't know to grab the blue bag by the oxygen tank and attach it to his trach and start bagging while calling for help. He wasn't responsive and then she ran and got us. We called 911 and they came. The whole issue with home nursing was that she was not prepared for an emergency, in this case, a mucus plug. [25]

Family caregivers felt they needed to speak up and challenge some healthcare professionals and paid carers over their competency and skills. Some described the need to be vigilant and supervise care at home, on the ward, and in paediatric intensive care to ensure no mistakes were made [23, 26]. Some felt less secure in hospital than at home [26] and felt the need to take on the role of the child's nurse, feeling unable to leave the child's bedside or sleep [27].

Others reported being unable to sleep because they could not trust the nurses or carers providing care in their home [4, 28, 29, 30], with one parent fitting a video camera to monitor overnight care following an error [4].

We had nurses in here who didn't know how to suction. Most of them didn't know how to change a trach… if you're sending a nurse to a child who needs suctioning… you'd think you'd send a nurse that knows how to suction… I mean I had nurses pushing me aside saying they know what to do and obviously they didn't. We can't go to sleep and close the door and trust the nurse. [29]

Trust was not only knowing if healthcare professionals and paid carers were physically able to care for the child, it was also about how they interacted with the child.

They mistreated her, and treated her like a robot. Every single time a nurse walked in the room, they treated her like she was not even there. [31]

For some family caregivers, previous bad experiences led to a growing mistrust of professionals and the wider system.

There was a time when I felt like I just want to take my child and go in the woods and take care of my child because I don't trust that anyone's going to take care of him properly. [32]

##### 3.2.2.2 Shared Decision‐Making and Respect for Families' Expertise

Family caregivers highlighted the importance of having their concerns and expertise listened to and acknowledged. Some felt that their ‘voice is lost*’* when interacting with services [5].

I expressed my frustration to every single professional that I was speaking to. Sometimes I felt as though my voice was just bouncing off bare walls and hitting me back. [5]

Family caregivers varied in how involved they wanted to be in decision‐making. For most it was important to feel ‘my opinion matters here’ [33] and like they were ‘part of the team’ [23] and ‘not labelled as difficult’ [34].

[Being] treated like an equal part of the team, that helps me with how to help the doctors do their job and how to help my son get better, when we're all working together… [Clinician] is an expert in complex care. [Clinician] is an expert in GI [gastrointestinal]. As a parent, I'm an expert on my son. [34]

They valued when healthcare professionals asked their views, and acknowledged gaps in their own knowledge. However, there needed to be a balance between parents/carers sharing their knowledge and not being expected to take over healthcare professionals' medical responsibility [26], with some family caregivers feeling that they were given too much responsibility, and required to direct their child's care [4, 32].

I realized sitting there in a pediatric hospital … that whether or not my son survived metabolic acidosis on a Saturday evening was on my shoulders. [32]

Family caregivers wanted to be kept informed and for professionals to explain things clearly [7]. Some described doing extensive research, for example by reading journal articles or using Facebook groups to learn from other families' experiences [7]. They wanted their expertise to be recognised and respected.

What does it hurt for a doctor to hand over a research paper that they've been just commenting from, or write out the terminology properly so that the parent can look it up. Yes, I get Dr. Google is a bad thing, but there's definitely some importance to that too, so that everybody's educated on making the best choices. [32]

It was especially important to family caregivers to feel they were in control of care in the home [35]. Family caregivers were often involved in training new carers and described the importance of carers listening to and learning from parental expertise, such as how to interpret the child's cues [29].

just be my support; don't be another source of stress…I would love your support, but you have to let me be the mother and let Rob be the father; let us make the decisions… [35].

Family caregivers wanted professionals to adapt their communication so the child could also understand and have a voice in decisions about their care where possible [26].

##### 3.2.2.3 Compassion and Understanding

Family caregivers wanted professionals to demonstrate humanness, understanding and empathy towards them [33, 36], for example, by showing empathy for their lack of sleep and subsequent mood swings [26]. Some felt that the staff determining eligibility for care packages in the home were ‘too far removed from the lived experience of caring for these children at home’ [7]. ‘Gestures of compassion’ could help ameliorate some of parents' trauma [32].

It was an NICU [neonatal intensive care] fellow. A really, really wonderful woman who shed a tear because it was the fifth or sixth day in a row that her and I needed to call [child's] dad and tell him the new developments. I said to her that day, “Can you tell him? I'm really tired of calling my man and breaking his heart all over again. I do not think I have it in me. I get that you think it's important that he knows this, but can you tell him?” And she shed a tear sitting there with me. Understanding what we were doing to [child's] dad as well. [32]

There was a perception that ‘quality‐of‐life assumptions’ were sometimes made by professionals who only saw the child at their sickest, which could sometimes affect medical decision‐making [31]. Family caregivers felt that some professionals had difficulty ‘understanding the child's best’ [26], and did not ‘value’ their child's life [37], which left them fearing for their child's safety.

They wanted healthcare professionals and paid carers, particularly in the home, to ‘truly care about their child*’* [7] and not *‘*think of it as a job*’* [29]. It was important to family caregivers that home nurses were flexible and fitted in with the needs of the child and family, and understood the difference between hospital and providing care in the home [35].

Family caregivers wanted truth and honesty from professionals [21, 38], a realistic view of the road ahead, and transparency about the challenges [21]. Some medical terms or phrases were triggering, for example, ‘failure to thrive’ [32]. It was important that difficult conversations were held in an appropriate (private) place [32].

Several studies described instances of discrimination in care [31, 37], in relation to disability, race and socioeconomic backgrounds [31, 37], which contributed to a growing mistrust of professionals, trauma and concerns for the child's safety [31, 32, 37].

The most common thing that we face is the disbelief of medical professionals about his pain … there is a stigma that comes from way, way back that Black people can endure more pain than other ethnicities, which is absolutely a myth. [31]

#### 3.2.3 Caring for the Whole Family

##### 3.2.3.1 Holistic Care for the Child and Family

Family caregivers wanted professionals to see the whole child and not view each issue separately [39]. They valued professionals/teams who approached the needs of the child holistically and saw ‘the big picture’ [33, 34, 39], and individualised care [32].

Family caregivers wanted professionals and policies to account for the emotional, financial and physical impact on the whole family [32], described as a ‘family‐centred approach to care’ [33]. They valued advocacy and support with adapting their homes or finding alternative housing [30]. Adaptations to the home were not always ‘future proofed for the children's safety, privacy and dignity needs as they grow older’ [11].

Another source of stress for family caregivers was limited availability of financial support [5]. They reported a lack of time to explore and apply for financial support, which often came with complex application processes [5, 7]. Family caregivers described various out‐of‐pocket costs which were not covered by government funding/grants [7, 27, 30].

When you have 24‐h nursing in your home, they go through toilet paper, paper towels, light bulbs. We do a lot of laundry. All of my bills are higher – water, electricity, all that kind of stuff – because of having the home health care. [7]

Many family caregivers had to give up paid employment [22, 30]. Some wanted to work but found that was not easily combined with income support they were receiving [38]. Family caregivers needed supportive employers who understood that they had to put their child's safety first.

We've been fortunate. I can't imagine what would happen if I ended up reporting to some other manager who sees my situation and goes, “I don't care. You still need to be here at such and such a time.” I can't. I don't have a nurse there at my house. I can't call a babysitter off the street to come watch my kid because they got to have a freaking medical degree. [laughs] That is a huge fear of mine. [7]

Family caregivers also expressed concern for the impact on siblings [4, 24, 28, 30, 40], and guilt over the lack of time they had to spend with them [4, 30]. Good‐quality respite enabled parents to give more attention to siblings and attend events/activities with siblings [28]. Family caregivers emphasised the importance of recognising the psychological impact on siblings [4, 24].

I know my teenage daughter definitely suffers from it [PTSD] cos she can't stand to look at [Name]'s tubes, she can't stand to be in the room when the children are crying. [4]

##### 3.2.3.2 Family Caregiver Trauma

Family caregivers emphasised the integrated nature of their own well‐being and that of their child: if they were not supported to look after themselves, they would not be able to safely look after their child.

All I want is that…it's been recognized that the parents, we are a holistic part of our child's wellbeing and if you do these little tweaks and help us more, it's helping the child as well and other families. [32]

Family caregivers described a sense of precarity, vulnerability and chronic stress [4, 26, 38].

It's like because you're living on the edge all the time, the littlest curve ball that is thrown into the mix just sends you over the edge. [4]

Many family caregivers wanted support from professionals/services for their own physical and mental health [5]. They felt that more quality respite or nursing care in the home would facilitate their sleep which would improve their emotional wellbeing and help them to deliver safer care for their child [38]. Many family caregivers felt mental health support was lacking and suggested it should be offered routinely to parents/carers [5, 30, 32], although some felt they had no time to attend to their own emotional needs [4, 38].

Hospital stays were often traumatic for the child and family [32]. Family caregivers wanted healthcare professionals to acknowledge their stress and trauma, to listen and validate their experiences.

Asking a mom, ‘How are you’, isn't enough. Eye contact, a sincere question, some time to listen, some validation, and some follow up … a little acknowledgement that what I'm trying to do is difficult would go a long, long way. [32]

Some family caregivers experienced a ‘strong sense of losing control in their lives’ [38] and a sense of grief and loss [38], exacerbated by uncertainty associated with their child's health [23]. Many felt isolated and that friends, family and professionals did not understand their reality [4, 22, 30]. The intensity of the child's medical needs often meant that daily life was dictated by medical routines [4, 22]. Some family caregivers also had to manage life‐threatening emergencies, and needed support to process this trauma.

By the time we got to the hospital [name of child] was so shut down they could only find a vein in her head [um] and she had to be resuscitated… she was so close to dying and I hadn't realised how close to dying she was … that was the single worst time of everything the whole lot and that's the memory you have when you're lying in bed thinking about your mind works overtime, that memory there, that makes me stop in my tracks. [4]

Family caregivers valued when professionals connected them with other families or organised peer support groups [21, 25, 27, 41]. Some provided informal peer support to others [25]. One study which focused on fathers' experiences emphasised that support is often more focused on the mother, and did not meet the needs of fathers, such as parent support groups which were not aimed at fathers [22]. Fathers' concerns in this study included providing intimate care for their children and barriers like accessing toilet and changing facilities particularly when caring for their daughters, and a lack of flexibility from employers for fathers.

##### 3.2.3.3 The Importance of Reliable Care in the Home

The decision to have nurses or paid carers in their home was often not easy for parents/carers, as it impacted many aspects of family life [7, 30, 35] and was often only accepted when critical for the safe care of their child. It was often difficult to find nurses and paid carers to fill care packages, and many experienced gaps in care at the last minute when shifts were not filled [7, 21, 30]. Some family caregivers were concerned about complaining about the quality of care in case the service reduced their hours [29], whilst others made formal complaints following concerns of harm [30]. Others resigned themselves to not having any care in the home due to problems with the reliability and safety of care.

When care in the home was not reliable, family caregivers were on duty 24‐7 for many consecutive days and nights. This affected their ability to work and could result in severe sleep deprivation, which posed a risk to the child's safety and affected the health of family caregivers [4, 28].

On one tube change occasion, I put in a smaller [tracheostomy] tube rather than the right tube size and didn't notice for a couple of hours [um] and I thought then, this is actually dangerous. I can't do this anymore. [4]

When family caregivers found a provider or person that they could trust, it made a big difference to their lives. Some felt that good quality care in the home reduced the frequency of hospital admissions [7].

My child adores her …. [The carer] knows [my child] probably just as well as we all do. We've had [this carer] for about 10 years, so she really does know our family and [my child]. I trust her with everything…she's been trained to do all that stuff…She knows the signs better than my husband that [our child] is getting unwell… [38]

#### 3.2.4 System Organisation

##### 3.2.4.1 Fragmented Systems

Across studies in multiple different countries, family caregivers reported needing to take on the role of care coordinator and advocate due to poor communication and coordination between professionals [4, 5, 32, 38, 39, 42]. This was described by some as a ‘full‐time job’ [39]. Poor care coordination posed a risk of harm to the child, including delayed care, missed medications and multiple surgeries which could have been combined [39, 40]. Family caregivers described a constant fight to navigate complex and fragmented systems [21], with excessive paperwork and poor communication between services [27, 39].

Most of my stress of having a medically ill child is not actually having a medically ill child, it's all of this other stuff I'm dealing with. Fighting [with] the insurance company, the doctors. Paperwork and phone calls and the pharmacy and prior authorizations. It's just a constant fight for everything. It's basically case management work. [7]

The task of navigating the system consumed enormous time and energy that could otherwise have been spent with their child, impacting their mental health and relationship with their partner [5, 38, 39].

I have never been able to feel like a mother. I feel more like a care coordinator. There are times when I look at my child and just go, “Oh my God, I miss being his mother.” I don't even think he knows I am his Mom. [39]

Family caregivers described learning to navigate the system over time through ‘trial and error’ [38], and developing an increasing assertiveness [7, 37, 38]. They fought and appealed decisions, particularly around care packages or home adaptations [7, 11, 39].

I had to file a fair hearing appeal on their [DHHS; Medicaid patient] decision on nursing hours at one point. …you're supposed to have a hearing and a decision within 90 days. We had to wait for a hearing well over 180 days. So I did contact the federal level of Medicaid…I also contacted the commissioner…it was a pretty complex scene, but I guess the biggest picture of that is that you have to be a strong advocate for your child and what has to be number one is your child's safety and your child's ability to live. [7]

Family caregivers felt that systems were not designed for children with medical complexity, and inflexible rules and regulations exacerbated problems [5, 23, 39]. Often services were not well informed about the services other agencies provided [39]. Coordinating appointments and short‐notice cancellations of appointments were frustrating for families [27, 38, 41]. Transition to adult services was another area of concern [7, 39, 41].

Difficulties accessing the required supplies, medication and medical equipment were common [4, 5, 7, 27, 37, 39, 43, 44]. Issues included receiving the wrong equipment; delays in deliveries; equipment breakages; having to reuse equipment which increased the infection risk; difficulties accessing critical medication; and being confined to their home as they were unable to obtain portable equipment.

Family caregivers reported that equipment suppliers and insurance companies were sometimes slow to address problems, with some unsure who to contact to resolve issues [7, 21, 30, 39]. Issues with insurance or payments meant that some family caregivers paid for supplies out‐of‐pocket [7, 27, 30]. Some sought assistance from other parents [25, 30], for example by using a Facebook group to help with tracheostomy supply issues, which worked like an ‘underground medical supply share’ and was ‘a lifesaver’ [25].

Hospital admissions were sometimes poorly coordinated, without anybody to help lead the care and coordinate across teams: there was a need for a long‐term ‘game plan’, and it was felt that the hospital was not the solution to this [43]. One parent described discharging themselves from hospital due to frustrations around a lack of coordination and a clear plan:

You keep changing doctors and reexplaining yourself, and nobody has a clear idea of what to do. … Last time, we were, like, ‘No, we're not gonna just stick around and wait’… because it was, like, going all over again, going through the same stuff. [43]

Family caregivers' ability to navigate the system varied greatly leading to inequalities in care. Those with limited English encountered issues like delayed or missed medications [44]. Family caregivers reported a lack of support to navigate the system or to fill out paperwork [30].

##### 3.2.4.2 System Design That Supports the Child and Family

Family caregivers valued the option of virtual appointments where appropriate [27, 33, 41, 45]. Travelling to multiple appointments was often expensive and logistically challenging, and getting time off work or arranging childcare was difficult. Sometimes a quick video call could help prevent hospital admissions [45]. Not everything could be done via phone/videocall (e.g. certain therapies, tests or scans). Where face‐to‐face appointments were needed, family caregivers valued having appointments closer to home, rather than having to travel long distances to specialist tertiary centres [33].

Making sweeping statements that it's always better to bring them into a clinic and that it's always better for a physician to physically see a patient is not helpful. The ultimate respect is to allow this option to exist. [41]

Continuity of professionals was important for family caregivers [5, 35, 38] and made them feel more secure [26]. Consistency helped improve communication, reduce repetition, and ensure care was individualized and safe [33].

I didn't want to lose Dr. [name]. It was me that insisted that we kept him. (P13) He [consultant] has been with us since before [child's name] was born, so we were quite keen that we didn't lose that connection. [5]

Family caregivers described a desire for a single clinician who was ‘in charge of their child's care’ [34] and having one number to call for problems [34]. Several studies described parents' experiences of specialist complex care clinics or dedicated care coordinators/key workers [5, 33, 34, 46]. Care coordinators were valued for their multifaceted roles which included: keeping different specialists up‐to‐date; advocating for families; mediating disagreements between parents and professionals; scheduling/coordinating appointments; providing emotional support to parents; and assisting with paperwork [5, 34]. Family caregivers appreciated having lead professionals to contact for support when they were having issues accessing medications, supplies or equipment [33]. Dedicated care coordinators could serve as interpreters for family caregivers with limited English, i.e. communicating with pharmacies, suppliers and home healthcare, and writing letters on behalf of parents to organisations such as immigration agencies [44].

Professionals who acted as key workers or care coordinators (e.g. children's community nurses) did not necessarily have ‘care coordinator’ in their job title or description [5]. Having a named ‘care coordinator’ was not always helpful, particularly when they were ‘tied’ to a specific health system [40]. One parent asked, ‘Do I have to coordinate the care coordinators too?’ [40]. Family caregivers did not always view care coordination as a cohesive responsibility/role, rather describing *‘*episodes when their child's care was coordinated or ‘in sync,’ and episodes when the care was clearly unsynchronized’ [40].

Multidisciplinary clinics or joint appointments were another helpful way of coordinating care and decision‐making [40], as were professionals who crossed organisational barriers, such as health professionals who did school visits [33]. Family caregivers appreciated when family support workers or charities helped with financial support [5]. Professionals who used rules, regulations and policies with flexibility to meet the needs of children and families were particularly valued [28, 32, 40].

What was nice during the pandemic is you could use that [respite] funding for a broad range of things not just respite and you didn't have to ﬁll out 5,000 forms to justify it during the pandemic. [27]

Family caregivers emphasised the importance of having quick access to specialists when there a problem, for example, by having ‘open access’ to their local children's ward [22] or having access to specialists out‐of‐hours that knew their child's history [34]. When good support from general practitioners (GPs) was in place, this was seen as valuable [22].

## 4 Discussion

### 4.1 Summary of Findings

This synthesis explores the experiences of 524 family caregivers of services for children with medical complexity. Their priority was their child's safety. They felt unable to trust some of the professionals caring for their child and didn't always feel their expertise was listened to. Family caregivers wanted professionals and services to provide holistic care and recognise the impact of the child's needs on the whole family. Navigating fragmented systems exacerbated their stress and safety concerns.

### 4.2 What This Study Adds

The World Health Organisation defines patient safety as‘the absence of preventable harm to a patient and reduction of risk of unnecessary harm associated with health care to an acceptable minimum.’ [47] Future research should define what safety means to family caregivers, as it may be that their definition of safety is slightly different to the WHO definition. The data in this review suggests it would include trying to keep their child healthy and well, and protect them from avoidable harm. Children with medical complexity rely on medical equipment and daily nursing care to keep them alive [4, 48], and this care is provided in many different settings including the home and school. These children need to be cared for by people with the skills to provide specialist medical care and to manage rapid deterioration and emergencies. They are particularly vulnerable to medical errors and harm [8, 9, 10, 49]. Family caregivers need to be able to trust the professionals caring for the child [50], and they go to great length to monitor the safety of their child's care in hospital and at home [4, 9, 51]. Family caregivers cannot be on duty 24‐7 to care for their child. Respite and nursing care in the home will only enable families to have a break, if they feel they can trust those caring for their child. The majority of the patient safety literature is focused on hospital care: home care has been underexplored in the field to‐date [52]. The safety of care across all settings, including hospitals, home and schools is an important focus for future interventions to improve care for children with medical complexity.

There is an extensive literature describing family caregivers' experiences of care for children with medical complexity, but the majority of this literature is focused on hospitals or nursing care in the home, and the evidence predominately comes from Canada and the United States. No studies were identified which focused on care in schools. Support from the social care sector or charitable organisations were mentioned in some studies, but no studies specifically focused on family caregivers' experiences of social care services. The boundary between what is the remit of health and what is the remit of social care is often not clear for children with disabilities [53]. In the education and social care literature, children with medical complexity are often grouped into the wider group of children with disabilities which includes children with autism, ADHD, diabetes and other common conditions: many of this group will not have medical complexity.

### 4.3 Implications for Practice

There needs to be a shift from providing care just to the child to providing care for the whole family: the child's medical needs mean that families have many social care needs, including finances, housing, sibling support etc, for which they need more support. There is a need for family‐centred care interventions [54]. Suggestions for service improvement from this synthesis include routine offering of support for parental/carer mental health, reducing the administrative burden of paperwork, providing support with coordination of care especially across sectors and giving families the option of virtual appointments where appropriate. Complex systems that are difficult to navigate and access risk exacerbating inequities in outcomes for children with medical complexity [14, 44]. Clearer lines of responsibility are needed across different services and sectors and these need to be openly shared with professionals and families. Comprehensive care programmes and care coordination services who take a lead on care and support with the nonmedical needs of the child and family are a growing area of research but the evidence base is mixed [16]. The safety of care is an important outcome measure for interventions.

### 4.4 Strengths and Limitations

We excluded studies which included a mixed sample of professionals and family caregivers and did not report separately on the family caregivers perspective to ensure the professional voice did not influence our findings. It was sometimes difficult to tell from the participant descriptions in the studies if they clearly met Cohen's definition of medical complexity: we may have missed some relevant studies if they did not use the term ‘medical complexity’ or did not provide enough information about the participants for us to tell if they met the definition. A lack of literature on education and social care meant that the synthesis is mostly focused on healthcare providers and settings. The majority of papers are from the United States and Canada, where the healthcare system is different from that in the United Kingdom and many European countries. Sampling meant that not all literature was synthesised, and some important experiences of families may have been missed. Two additional studies from the United Kingdom were included to improve the representation of studies from this country but this meant that some lower quality studies were included. The voice of fathers was underrepresented and needs further exploration [22, 55].

## 5 Conclusions

The existing literature focuses primarily on care in hospitals and at home. There is a significant gap in knowledge about parents'/carers' experiences of social care and education services. Family caregivers' primary concern is keeping their child safe across all settings of care and protecting them from avoidable harm. Not feeling listened to or involved in decision‐making contributes to family caregivers' concerns for their child's safety. Holistic support is needed to address the needs of the whole family. Interventions are needed to improve coordination and lines of responsibility between different services and sectors to reduce the onus on parents to navigate a complex and fragmented systems. The safety of care is an important outcome measure for interventions to improve care. Future work needs to define exactly what family caregivers mean by safety.

## Author Contributions

**Bethan Page:** conceptualisation, writing – original draft, writing – review and editing, formal analysis, methodology, data curation, project administration, funding acquisition. **Pru Holder:** formal analysis, writing – review and editing, methodology, data curation. **Katie Doidge:** writing – review and editing, writing – original draft, methodology, data curation. **Lisa Hinton:** conceptualisation, writing – review and editing, methodology. **Lorna K. Fraser:** conceptualisation, writing – review and editing, methodology, formal analysis.

## Ethics Statement

Ethical approval was not required for this review as data used for analysis were extracted from published studies and the parent workshop was classed as PPI activities.

## Conflicts of Interest

The authors declare no conflicts of interest.

## Supporting information


**Supplementary File 1. Example Search Strategy: Medline**.

**Supplementary File 2. Extracted papers not included in thematic synthesis (n=42)**.

**Supplementary File 3. CASP Quality Checklist**.

## Data Availability

Data sharing is not applicable to this article as no datasets were generated or analysed during the current study.

## References

[1] S. Jarvis , G. Richardson , K. Flemming , and L. K. Fraser , “Numbers, Characteristicsm, and Medical Complexity of Children With Life‐Limiting Conditions Reaching Age of Transition to Adult Care in England: A Repeated Cross‐Sectional Study,” NIHR Open Research. 2 (April 2022): 27. 35923178 10.3310/nihropenres.13265.1 PMC7613215

[2] E. Cohen , D. Z. Kuo , R. Agrawal , et al., “Children With Medical Complexity: An Emerging Population for Clinical and Research Initiatives,” Pediatrics 127 (2011): 529–538. 21339266 10.1542/peds.2010-0910 PMC3387912

[3] K. Millar , C. Rodd , G. Rempel , E. Cohen , K. M. Sibley , and A. Garland , “The Clinical Definition of Children With Medical Complexity: A Modified Delphi Study,” Pediatrics 153, no. 6 (June 2024): e2023064556. 38804054 10.1542/peds.2023-064556

[4] B. F. Page , L. Hinton , E. Harrop , and C. Vincent , “The Challenges of Caring for Children Who Require Complex Medical Care at Home: ‘The Go Between for Everyone Is the Parent and as the Parent That's an Awful Lot of Responsibility,” Health Expectations 23, no. 5 (October 2020): 1144–1154. 32542954 10.1111/hex.13092 PMC7696130

[5] E. V. McLorie , J. Hackett , and L. K. Fraser , “Understanding Parents' Experiences of Care for Children With Medical Complexity in England: A Qualitative Study,” BMJ Paediatrics Open 7, no. 1 (August 2023): e002057. 37550084 10.1136/bmjpo-2023-002057 PMC10407344

[6] K. Dybwik , T. Tollåli , E. W. Nielsen , and B. S. Brinchmann , “Fighting the System: Families Caring for Ventilator‐Dependent Children and Adults With Complex Health Care Needs at Home,” BMC Health Services Research 11 (2011): 156. 21726441 10.1186/1472-6963-11-156 PMC3146406

[7] R. D. Boss , J. C. Raisanen , K. Detwiler , et al., “Lived Experience of Pediatric Home Health Care Among Families of Children With Medical Complexity,” Clinical Pediatrics 59, no. 2 (February 2020): 178–187. 31849237 10.1177/0009922819894006

[8] B. Page , R. Nawaz , S. Haden , C. Vincent , and A. C. H. Lee , “Paediatric Enteral Feeding at Home: An Analysis of Patient Safety Incidents,” Archives of Disease in Childhood 104, no. 12 (December 2019): 1174–1180. 31201158 10.1136/archdischild-2019-317090 PMC6900243

[9] S. Mauskar , T. Ngo , H. Haskell , et al., “In Their Own Words: Safety and Quality Perspectives From Families of Hospitalized Children With Medical Complexity,” Journal of Hospital Medicine 18, no. 9 (September 2023): 777–786. 37559415 10.1002/jhm.13178 PMC11088437

[10] R. F. Nawaz , B. Page , E. Harrop , and C. A. Vincent , “Analysis of Paediatric Long‐Term Ventilation Incidents in the Community,” Archives of Disease in Childhood 105, no. 5 (May 2020): 446–451. 31848150 10.1136/archdischild-2019-317965 PMC7212935

[11] T. K. Mitchell , L. Bray , L. Blake , A. Dickinson , and B. Carter , “‘I Feel Like My House Was Taken Away From Me’: Parents' Experiences of Having Home Adaptations for Their Medically Complex, Technology‐Dependent Child,” Health & Social Care in the Community 30, no. 6 (November 2022): e4639–e4651. 35715967 10.1111/hsc.13870 PMC10083937

[12] V. Fisher , K. Atkin , and L. K. Fraser , “The Health of Mothers of Children With a Life‐Limiting Condition: A Qualitative Interview Study,” Palliative Medicine 36, no. 9 (October 2022): 1418–1425. 36113084 10.1177/02692163221122325 PMC9597138

[13] N. A. Murphy , J. Alvey , K. J. Valentine , K. Mann , J. Wilkes , and E. B. Clark , “Children With Medical Complexity: The 10‐Year Experience of a Single Center,” Hospital Pediatrics 10, no. 8 (August 2020): 702–708. 32699000 10.1542/hpeds.2020-0085

[14] C. Breen , L. Altman , J. Ging , M. Deverell , S. Woolfenden , and Y. Zurynski , “Significant Reductions in Tertiary Hospital Encounters and Less Travel for Families After Implementation of Paediatric Care Coordination in Australia,” BMC Health Services Research 18, no. 1 (October 2018): 751. 30285821 10.1186/s12913-018-3553-4 PMC6171181

[15] E. Cohen , S. Quartarone , J. Orkin , et al., “Effectiveness of Structured Care Coordination for Children With Medical Complexity: The Complex Care for Kids Ontario (CCKO) Randomized Clinical Trial,” JAMA Pediatrics 177, no. 5 (May 2023): 461–471. 36939728 10.1001/jamapediatrics.2023.0115 PMC10028546

[16] A. R. Harvey , E. Meehan , N. Merrick , et al., “Comprehensive Care Programmes for Children With Medical Complexity,” Cochrane Database of Systematic Reviews no. 5 (2024): CD013329, 10.1002/14651858.CD013329.pub2. 38813833 PMC11137836

[17] A. Tong , K. Flemming , E. McInnes , S. Oliver , and J. Craig , “Enhancing Transparency in Reporting the Synthesis of Qualitative Research: ENTREQ,” BMC Medical Research Methodology 12 (2012): 181. 23185978 10.1186/1471-2288-12-181 PMC3552766

[18] H. Ames , C. Glenton , and S. Lewin , “Purposive Sampling in a Qualitative Evidence Synthesis: A Worked Example From a Synthesis on Parental Perceptions of Vaccination Communication,” BMC Medical Research Methodology 19, no. 1 (January 2019): 26. 30704402 10.1186/s12874-019-0665-4 PMC6357413

[19] Critical Appraisal Skills Programme . CASP Qualitative Checklist (2018), https://casp-uk.net/casp-tools-checklists/qualitative-studies-checklist/.

[20] J. Thomas and A. Harden , “Methods for the Thematic Synthesis of Qualitative Research in Systematic Reviews,” BMC Medical Research Methodology 8 (2008): 45. 18616818 10.1186/1471-2288-8-45 PMC2478656

[21] L. G. Amar‐Dolan , M. H. Horn , B. O'Connell , et al., “‘This Is How Hard It Is’ Family Experience of Hospital‐to‐Home Transition With a Tracheostomy,” Annals of the American Thoracic Society 17, no. 7 (July 2020): 860–868. 32267725 10.1513/AnnalsATS.201910-780OC PMC7328176

[22] L. Hobson and J. Noyes , “Fatherhood and Children With Complex Healthcare Needs: Qualitative Study of Fathering, Caring and Parenting,” BMC Nursing 10 (April 2011): 5. 21496238 10.1186/1472-6955-10-5 PMC3094306

[23] J. E. Rennick , I. St‐Sauveur , A. M. Knox , and M. Ruddy , “Exploring the Experiences of Parent Caregivers of Children With Chronic Medical Complexity During Pediatric Intensive Care Unit Hospitalization: An Interpretive Descriptive Study,” BMC Pediatrics 19, no. 1 (August 2019): 272. 31387555 10.1186/s12887-019-1634-0 PMC6683527

[24] S. Thomas and M. Price , “Respite Care in Seven Families With Children With Complex Care Needs,” Nursing Children and Young People 24, no. 8 (2012): 24–27. 10.7748/ncyp2012.10.24.8.24.c9338 23167016

[25] J. Sherman , K. L. Bower , and K. Eskandanian , “‘100 Things I Wish Someone Would Have Told Me’: Everyday Challenges Parents Face While Caring for Their Children With a Tracheostomy,” Qualitative Health Research 34 (September 2024): 1108–1118. 38193439 10.1177/10497323231217387 PMC11492094

[26] M. Hagvall , M. Ehnfors , and A. Anderzén‐Carlsson , “Experiences of Parenting a Child With Medical Complexity in Need of Acute Hospital Care,” Journal of Child Health Care 20, no. 1 (March 2016): 68–76. 25352538 10.1177/1367493514551308

[27] V. C. Fong , J. Baumbusch , and K. Basu Khan , “‘A Very Different Place From When the Pandemic Started’: Lessons Learned for Improving Systems of Care for Families of Children With Medical Complexity,” Journal of Child Health Care 29, no. 2 (2023): 339–352. 37740509 10.1177/13674935231203274 PMC12145465

[28] R. Welsh , S. Dyer , D. Evans , and J. Fereday , “Identifying Benefits and Barriers to Respite for Carers of Children With Complex Health Needs: A Qualitative Study,” Contemporary Nurse 48, no. 1 (May 2014): 98–108. 25410200 10.1080/10376178.2014.11081931

[29] K. Keilty , D. Nicholas , and E. Selkirk , “Experiences With Unregulated Respite Care Among Family Caregivers of Children Dependent on Respiratory Technologies,” Journal of Child Health Care 22, no. 1 (March 2018): 46–56. 29278917 10.1177/1367493517746770

[30] C. C. Foster , S. Shaunfield , L. E. Black , P. Z. Labellarte , and M. M. Davis , “Improving Support for Care at Home: Parental Needs and Preferences When Caring for Children With Medical Complexity,” Journal of Pediatric Health Care 36, no. 2 (March 2022): 154–164. 34688541 10.1016/j.pedhc.2020.08.005

[31] S. G. Ames , R. K. Delaney , A. J. Houtrow , et al., “Perceived Disability‐Based Discrimination in Health Care for Children With Medical Complexity,” Pediatrics 152, no. 1 (2023): e2024068782. 10.1542/peds.2022-060975 37357731

[32] T. Dewan , K. Birnie , J. Drury , et al., “Experiences of Medical Traumatic Stress in Parents of Children With Medical Complexity,” Child: Care, Health and Development 49, no. 2 (March 2023): 292–303. 35947493 10.1111/cch.13042 PMC10087969

[33] O. Hlyva , C. Rae , S. Deibert , et al., “A Mixed‐Methods Feasibility Study of Integrated Pediatric Complex Care: Experiences of Parents With Care and the Value of Parent Engagement in Research,” Frontiers in Rehabilitation Sciences. 2 (2021): 2. 10.3389/fresc.2021.710335 PMC9397898 36188846

[34] J. A. Yu , S. Cook , C. Imming , et al., “A Qualitative Study of Family Caregiver Perceptions of High‐Quality Care at a Pediatric Complex Care Center,” Academic Pediatrics 22, no. 1 (January 2022): 107–115. 34020106 10.1016/j.acap.2021.05.012 PMC9979253

[35] M. Mendes , “Parents' Descriptions of Ideal Home Nursing Care for Their Technology Dependent Child, Themselves, and Their Families,” Journal of Pediatric Nursing 39, no. 2 (2017): 91–96. 23705300

[36] B. V. Houlihan , C. Coleman , D. Z. Kuo , B. Plant , and M. Comeau , “What Families of Children With Medical Complexity Say They Need: Humanism in Care Delivery Change,” supplement, Pediatrics 153, no. S1 (January 2024): e2023063424F. 10.1542/peds.2023-063424F 38165241

[37] S. G. Ames , R. K. Delaney , C. Delgado‐Corcoran , et al., “Impact of Disability‐Based Discrimination in Healthcare on Parents of Children With Medical Complexity,” Developmental Medicine & Child Neurology 66, no. 9 (September 2024): 1226–1233. 38327250 10.1111/dmcn.15870 PMC11579817

[38] A. Moyes , T. Abbott , S. Baker , C. Reid , R. Thorne , and E. Mörelius , “A Parent First: Exploring the Support Needs of Parents Caring for a Child With Medical Complexity in Australia,” Journal of Pediatric Nursing 67 (November 2022): e48–e57. 36192287 10.1016/j.pedn.2022.09.018

[39] S. L. Golden and S. Nageswaran , “Caregiver Voices: Coordinating Care for Children With Complex Chronic Conditions,” Clinical Pediatrics 51, no. 8 (August 2012): 723–729. 22563062 10.1177/0009922812445920

[40] R. Cady and J. Belew , “Parent Perspective on Care Coordination Services for Their Child With Medical Complexity,” Children 4, no. 6 (June 2017): 45. 28587274 10.3390/children4060045 PMC5483620

[41] V. C. Fong , J. Baumbusch , and K. Khan , “‘Can You Hear Me Ok?’: Caregivers of Children With Medical Complexity and Their Perspectives of Virtual Care During COVID‐19,” Journal of Pediatric Health Care 38, no. 1 (January 2024): 30–38. 37725030 10.1016/j.pedhc.2023.08.008

[42] F. Buchanan , C. Lai , E. Cohen , G. Milo‐Manson , and A. Shachak , “Decision‐Making for Parents of Children With Medical Complexities: Activity Theory Analysis,” Journal of Participatory Medicine 14, no. 1 (January 2022): e31699. 35037890 10.2196/31699 PMC8804956

[43] J. C. Leary , R. Krcmar , G. H. Yoon , K. M. Freund , and A. M. LeClair , “Parent Perspectives During Hospital Readmissions for Children With Medical Complexity: A Qualitative Study,” Hospital Pediatrics 10, no. 3 (2020): 222–229. 32029432 10.1542/hpeds.2019-0185 PMC7041550

[44] S. Nageswaran , M. B. Ellis , and M. S. Beveridge , “Communication Challenges Faced by Spanish‐Speaking Caregivers of Children With Medical Complexity: A Qualitative Study,” Journal of Racial and Ethnic Health Disparities 9, no. 6 (December 2022): 2218–2226. 34595676 10.1007/s40615-021-01161-x PMC8483426

[45] J. M. Frush , D. Y. Ming , N. Crego , et al., “Caregiver Perspectives on Telemedicine for Postdischarge Care for Children With Medical Complexity: A Qualitative Study,” Journal of Pediatric Health Care 37, no. 4 (July 2023): 356–363. 36670018 10.1016/j.pedhc.2022.12.009 PMC10330386

[46] G. Currie , D. Materula , N. Gall , et al., “Care Coordination of Children With Neurodevelopmental Disabilities and Medical Complexity During the COVID‐19 Pandemic: Caregiver Experiences,” Child: Care, Health and Development 49, no. 5 (September 2023): 834–845. 37407028 10.1111/cch.13149

[47] World Health Organisation . “Patient Safety [Internet].” 2024, https://www.who.int/news-room/fact-sheets/detail/patient-safety.

[48] I. Ten Haken , S. Ben Allouch , and W. H. Van Harten , “The Use of Advanced Medical Technologies at Home: A Systematic Review of the Literature,” BMC Public Health 18, no. 1 (February 2018): 284. 29482550 10.1186/s12889-018-5123-4 PMC6389044

[49] C. A. Schindler , E. S. Pordes , S. D. Finkenbinder , and K. J. Lee , “Safety in Children With Medical Complexity: Our Canaries in the Coal Mine?,” Current Treatment Options in Pediatrics 5 (2019): 165–182.

[50] T. Dewan , A. Whiteley , L. J. MacKay , et al., “Trust of Inpatient Physicians Among Parents of Children With Medical Complexity: A Qualitative Study,” Frontiers in Pediatrics 12 (September 2024): 1443869. 39398419 10.3389/fped.2024.1443869 PMC11466756

[51] S. Mitchell , J. L. Spry , E. Hill , J. Coad , J. Dale , and A. Plunkett , “Parental Experiences of End of Life Care Decision‐Making for Children With Life‐Limiting Conditions in the Paediatric Intensive Care Unit: A Qualitative Interview Study,” BMJ Open 9, no. 5 (May 2019): e028548. 10.1136/bmjopen-2018-028548 PMC6528052 31072863

[52] A. W. Wu , C. Vincent , J. Øvretveit , et al., “Gaps in Patient Safety: Areas That Need Our Attention,” Journal of Patient Safety and Risk Management 28 (2023): 246–252.

[53] Law Commission “Law Commission – Disabled Children's Social Care: Summary of the Consultation Paper.” https://lawcom.gov.uk/project/.

[54] A. J. Chow , A. Saad , Z. Al‐Baldawi , et al., “Family‐Centred Care Interventions for Children With Chronic Conditions: A Scoping Review,” Health Expectations: An International Journal of Public Participation in Health Care and Health Policy 27, no. 1 (2024): e13897, 10.1111/hex.13897. 39102737 PMC10837485

[55] V. Fisher , L. Fraser , and J. Taylor , “Experiences of Fathers of Children With a Life‐Limiting Condition: A Systematic Review and Qualitative Synthesis,” BMJ Supportive and Palliative Care 13 (2023): 15–26. 10.1136/bmjspcare-2021-003019 PMC9985706 34140322

