# Supplementary material for: Family Caregivers' Experiences of Services for Children With Medical Complexity: A Systematic Review and Qualitative Evidence Synthesis
Source: Health Expect. 2025 Sep 29;28(5):e70452. doi: 10.1111/hex.70452 (PMC12480434; doi:10.1111/hex.70452)
Supplement: Supplementary file 1 — Example Search Strategy: Medline. [file HEX-28-e70452-s003.docx]

**Supplementary File 1**

**Example Search Strategy: Medline**

Ovid MEDLINE(R) ALL <1946 to March 13, 2024>

1 focus groups/ or interviews as topic/ or narration/ or qualitative research/ or ((face or f2f or guided or depth or indepth or informal or semistructured or structured or unstructured) adj4 (discussion* or interview* or questionnaire*)).ti,ab,kf. or (ethnograph* or (field adj1 work) or fieldwork or (focus adj1 (group or groups)) or (key adj1 (informant or informants)) or qualitative).ti,ab,kf. 557988

2 Parent*.tw. or exp Parents/ 570300

3 Father*.tw. or exp Fathers/ 54082

4 Mother*.tw. or exp Mothers/ 281847

5 (caregiver* or carer*).tw. or exp Caregivers/ 123941

6 Family.tw. 969373

7 Families.tw. 301799

8 2 or 3 or 4 or 5 or 6 or 7 1858971

9 experiences.tw. 286488

10 Perspective*.tw. 478584

11 perception*.tw. 341659

12 views.tw. 96294

13 Medical* complex*.tw. 2921

14 Medica* fragil*.tw. 304

15 Technolog* dependen*.tw. 486

16 (complex* adj5 condition*).tw. 21723

17 Healthcare technolog*.tw. 826

18 Complex medical care.tw. 95

19 complex medical need*.tw. 188

20 (complex health* adj2 need*).tw. 776

21 13 or 14 or 15 or 16 or 17 or 18 or 19 or 20 26856

22 1 or 9 or 10 or 11 or 12 1475167

23 8 and 21 and 22 1022

24 limit 23 to (english language and yr="2011 -Current") 864
