# Supplementary material for: Family Caregivers' Experiences of Services for Children With Medical Complexity: A Systematic Review and Qualitative Evidence Synthesis
Source: Health Expect. 2025 Sep 29;28(5):e70452. doi: 10.1111/hex.70452 (PMC12480434; doi:10.1111/hex.70452)
Supplement: Supplementary file 2 — Extracted papers not included in thematic synthesis (n=42). [file HEX-28-e70452-s002.docx]

**Supplementary File 2**

**Extracted papers not included in thematic synthesis (n=42)**

| **Authors** | **Year of publication** | **Country** | **Setting** | **Aims** | **Methodology** | **Condition of children** | **Age of children** | **Parent participants** | **Analysis methodology** |
| --- | --- | --- | --- | --- | --- | --- | --- | --- | --- |
| Abebe et al., | 2020 | USA | All health and care settings | To describe medication management work, its constraints, and complexities from the perspectives of family caregivers of children with medical complexity | Observations of clinics, semi-structured interviews and reviews of program documents | Not reported | Range = 6 months - 17 years | Interviewed family caregivers of 11 different CMC.  Male caregiver, n = 1 Female, n = 10 Grandmother, n = 1 | Thematic analysis and Cognitive Work  Analysis (CWA) |
| An et al., | 2023 | South Korea | Hospital | To explore the experiences of South Korean mothers of their children with medical complexity under long-term hospitalization. | Semi-structured interviews | Mixed conditions | Range = 4 years - 18 years | Mothers, n = 7 | Thematic analysis |
| Baird et al., | 2016 | USA | Paediatric Intensive Care | To explore the delivery of continuity of nursing care in the PICU from the perspective of both parents and nurses. | Observations of clinics, semi-structured interviews and reviews of program documents | Mixed conditions | < 1 year, n = 2 1 - 5 years, n=2 6 - 10 years, n=2 11 - 15 years, n=1 | Mothers, n = 5 Fathers, n = 2 Nurses, n = 2 | Situational analysis |
| Barone et al., | 2020 | USA | Transition from hospital to home | To test the feasibility of Photovoice methodology to increase discharging clinician awareness of the environmental context and challenges in caring for children with CCI at home. We also aimed to identify existing discharge planning limitations for these children and their families. | Photovoice methodology | Mixed conditions | Infants | 12 families  Fathers, n = 2 | Thematic and interpretive analysis |
| Barton et al., | 2021 | USA | Home | To identify and describe workarounds families have developed to optimize medical device use for their needs. | Contextual inquiry interviews, semi-structured interviews, observation of notes and photographs | Not recorded | Range = 1 - 19 years | 30 caregivers  Female, n = 24 | Qualitative content analysis |
| Batson et al., | 2022 | USA | Home- focus on transportation | To explore the family experience and associated challenges of transporting children with medical complexity (CMC) in personal vehicles. | Semi-structured interviews | Mixed conditions | < 6 years, n = 6 6 - 11 years, n = 12 12 - 17 years, n = 8 ≥ 18 years, n = 13 | 29 participants  Female, n = 26 | Conventional content analysis |
| Black et al., | 2022 | USA | Home | To explore parent perceptions of barriers to and facilitators of mobility and accessibility of CMC in their home environment and the consequential impact on the lives of these children and their families. | Semi-structured interviews | Mixed conditions | Range = 2 - 18 years | Mothers, n = 16 Fathers, n = 2 | Thematic analysis |
| Bogetz et al., | 2021 | USA | All health and care settings (recruited via hospital) | To examine bereaved parents’ perspectives about supportive clinical care strategies among the complex chronic conditions parent population throughout their child’s medical journey and at end of life. | Cross-sectional survey | Mixed conditions | Median age at death = 11.1 years IQR = 1.9 - 20.3 years | Participants, n = 105 Mothers, n = 83 | Thematic analysis |
| Castro-Codesal et al., | 2024 | Canada | All health and care settings (recruited via hospital) | To gain an understanding of significant aspects of children’s tracheostomy journey from their parents’ perspectives. | Focus groups | Mixed conditions | Age at tracheostomy:  < 30 days, n = 1 30 - 90 days, n = 3 3 - 6 months, n = 6 6 - 12 months, n = 1 1 - 10 years, n = 0 10 - 18 years, n=1 | Female, n = 12 | Constructivist grounded theory |
| Cavicchioli Okido et al., | 2016 | Brazil | Pharmacy | How do mothers experience the pharmaceutical care of their technology-dependent children? | Open interviews and structured questionnaires | Not recorded | Not recorded.   The time caring for the children ranged from 18 months to 11 years | Mothers, n = 12 | Inductive content analysis |
| D'Aprano et al., | 2020 | Australia | Care coordination service | To determine what caregivers value most about the complex care service at the Royal Children's Hospital and explore caregiver perceptions of care | Mixed-methods survey | Mixed conditions | < 1 year, n = 4 1 - 4 years, n = 28 5 - 9 years, n = 16 10 - 14 years, n = 4 15 - 18 years, n = 0 > 18 years, n = 1 | Mothers, n = 47 Fathers, n = 2 Other, n = 2 Missing, n = 2 | Thematic analysis |
| deLima et al., | 2015 | Brazil | All health and care services | To highlight family strengths and weaknesses in terms of the care provided to children who are dependent on technology, and changes occurring following the return of these children to their homes; to discuss challenges and opportunities for family inclusion in the healthcare process. | Semi-structured  interviews and observation, symbolic interactionism | Mixed conditions | Range = 5 months - 7 years | Mothers, n = 13 Grandmothers, n = 1 | Content analysis |
| Giambra et al., | 2017 | USA | Hospital | The purpose of this qualitative research study was to expand our understanding of the process of communication between parents of hospitalized technology dependent children and their nurses originally detailed in the Theory of Shared Communication (TSC). | Semi-structured interviews | Mixed conditions | 4 - 7 years, n = 3 8 - 10 years, n = 1 11 - 15 years, n=1 | Mothers, n = 3 Fathers, n = 2 Nurses, n = 9 | Comparative analysis |
| Gold et al., | 2020 | USA | Hospital discharge | The objective of this study is to describe parent perspectives and priorities regarding discharge medication education for CMC. | Focus groups | Not reported | Not reported | 24 participants | Thematic analysis |
| Hess et al., | 2024 | USA | Complex care service at hospital | To explore family satisfaction and perceived quality of care in a pediatric neuromuscular care clinic to assess the value of the multidisciplinary clinic (MDC) model in delivering coordinated care to children with neuromuscular disorders, such as cerebral palsy | In-depth individual  interviews using a structured interview guide | Mixed conditions | Age at enrolment: 0 - 4 years, n = 15 (68.2%) 5 - 12 years, n = 2 (9.1%) 13 - 18 years, n = 5 (22.7%) | Mothers, n = 17 Fathers, n = 5 | Thematic analysis |
| Hirt et al., | 2023 | USA | All health and care services | To explore caregivers’ current and holistic experiences of caregiving for children with medical complexity and their perceptions of their unmet medical and psychosocial needs. | In-depth interview | Mixed conditions | 0 - 3 years, n = 4 (21.1%) 3 - 6 years, n = 7 (36.8%) 6 - 9 years, n = 1 (5.3%) 9 - 12 years, n = 2 (10.5%) 12 - 15 years, n = 4 (21.1%) 15 - 18 years, n = 1 (5.3%) | 19 caregivers   Female, n = 18 Male, n = 1 | Psychological  phenomenology |
| Keim-Malpass | 2023 | USA | All health and care services | To explore the structures and processes of family management among caregivers of children with medical complexity, with a focus on the underlying dynamic nature of family management practices and the role of members of their social network. | Interviews | Mixed conditions | Not mentioned | 20 caregivers,  Female, n = 18 Male, n = 2 | Responses were  analysed using constructivist grounded theory and situational analysis |
| Kellom et al., | 2023 | USA | All health and care services- focus on parental employment and finances | To better understand caregiving obligations of parents of CMC, availability and appropriateness of family medical leave act (FMLA) in facilitating their dual role as caregiver and employee, as well as ways in which this dual role affected caregivers’ employment stability and economic security. | Semi-structured interviews | Not reported | Range = 1 - 14 years Mean = 7 years | Participants, n = 16  Mothers, n = 16 (100%) | Thematic analysis |
| Kieren et al., | 2023 | USA | Hospital | To describe the process of identifying and reporting inpatient safety concerns from the perspective of parents of children with medical complexity (CMC). | Semi-structured interviews | Not reported | Not reported | Participants, n = 31  Female, n = 26 Male, n = 5 | Secondary analysis of transcript data from parent interviews conducted for the FACES study.  Inductive thematic analysis |
| Krieg et al., | 2023 | USA | Hospital | To identify coping factors for caregivers of children with medical complexity (CMC) to manage the stressors and experience associated with their child’s hospitalization. | Semi-structured interviews | Not reported | Range = 2 - 16 years | Caregivers, n = 14  Mothers, n = 11 Fathers, n = 3 | Qualitative analysis |
| Lin et al., | 2020 | USA | All health and care services | To describe parent perspectives of shared decision-making (SDM) for CMC and identify opportunities to improve elements of SDM specific to this vulnerable population. | Semi-structured  interviews | Mixed conditions | Range = 1 - 11 years | 32 parents  Female, n = 27 | Modified grounded  theory |
| Lindahl et al., | 2013 | Sweden | Home | To understand the meanings that parents had about the support they received from health care professionals who offered care for their ventilator-assisted child in the family home. | In-depth interviews | Mixed conditions | 3 years, n = 1 4 years, n = 1 8 years, n = 2 23 years, n = 1 | 10 parents 5 interviews  Fathers, n = 5 Mothers, n = 5 | Naïve understanding,  structural analyses, and comprehensive understanding. |
| Lord et al., | 2020 | Canada | All health and care services (focus on end of life) | To explore the experiences of bereaved family caregivers with advanced care planning (ACP) for CMC. | Semi-structured  interviews | Mixed conditions | Child's age at death: < 1 year, n = 1 (8%) 1 - < 5 years, n = 4 (33%) 5 - 10 years, n = 4 (33%) > 10 years, n = 3 (25%) | 13 parents  12 interviews  Mothers, n = 12 Fathers, n = 1 | Thematic analysis with an inductive approach |
| MacKay et al., | 2021 | Canada | Hospital | To present an account of parental experiences as they provided care for their hospitalized medically fragile infant | Semi-structured interviews | Mixed conditions | less than 1  year corrected age.  Mean gestational age = 33 weeks SD = 5.79 weeks Range = 23 - 41 weeks | 19 interviews  Mothers, n = 15 Fathers, n = 6 | Charmazian grounded theory: initial coding, focused coding, theorizing |
| Mauskar et al., | 2023 | USA | Hospital | To explore safety reports from families of hospitalized CMC to identify areas to improve safety/quality. | Survey | Mixed conditions | Mean = 14 years SD = 7 years | Parent, n = 190 (92.7) Grandparent, n = 4 (2%) Guardian, n = 7 (3.4%) Foster parent, n = 3 (1.5%) Other, n = 1 (0.5%)  Female, n = 168 (82%) Male, n = 37 (18%) | Secondary analysis, thematic analysis |
| Miller et al., | 2022 | USA | Hospital | To explore new parent awareness of health care costs, desire to discuss costs with clinicians, and impact of costs on parents’ medical decision-making. | Semi-structured  interviews and surveys | Mixed conditions | Two groups of criteria: Infants ≥14 days old currently in the NICU, and infants <1 year old seen in the NICU follow-up clinic | Families, n = 27 | Conventional content  analysis for interviews, descriptive analysis for surveys. |
| Ming et al., | 2019 | USA | Complex care service at hospital | To evaluate feasibility of mobile complex care plans (MCCPs) for CMC enrolled in a complex care program and to study MCCPs’ impact on parent engagement, parent experience, and care coordination. | Semi-structured interviews | Not reported. | Median = 7.7 years Minimum = 0.4 years Maximum = 18.5 years | Participants, n = 50  Female, n = 22 (45%) Male, n = 27 (55%) | Descriptive statistics  and conducted analyses in SAS |
| Morse et al., | 2021 | USA | All health and care services | (1) To ascertain parents’ perceived characteristics of child pain experiences (i.e., pain antecedents, behaviours, frequency, relief measures), (2) to determine the extent to which parents feel that caregivers adequately address pain, and (3) identify ways in which pain collaboration between parents and caregivers may be improved | Individual interviews | Not reported | Mean = 15 years Range = 2 - 33 years | 29 interviews  Mothers, n = 25 Fathers, n = 2 Mother-father pairs, n = 2 | Conventional and  directed approaches to qualitative content analysis. |
| Nageswaran et al., | 2024 | USA | Virtual care/telemedicine | To understand the benefits and challenges of telehealth from the perspectives of caregivers of CMC. | Semi-structured interviews | Mixed conditions | Median = 11 years Range = 3-17 years | 23 caregivers | Thematic content analysis |
| Nelson et al., | 2016 | USA | Hospital | To understand perspectives of parents of CMC enrolled in the Pediatric Medical Home Program (PMHP), a complex care program at University of California, Los Angeles (UCLA), about their decisions to bring their children in for acute care encounters that ultimately led to hospitalizations | Semi-structured  interviews | Mixed conditions | < 5 years, n = 14 (40%) 5 - 13 years, n = 16 (46%) 13 - 18 years, n = 5 (14%) | 35 parents  Mothers, n = 32 Fathers, n = 3 | Qualitative analysis |
| Norton et al., | 2022 | USA | Hospital discharge | To describe the process of mothers’ transition to home from the NICU caring for technology-dependent infants during the immediate post discharge period, their first 2 weeks at home, and a high-risk time for home caregiving errors related to appointments, medication administration, feedings, and equipment management. | Semi-structured interviews | Mixed conditions | Gestational age at birth: Mean = 27 weeks Range = 23 - 36 weeks | Mothers, n = 8 | Inductive qualitative  content analysis |
| Pitch et al., | 2023 | Canada | All health and care services (focus on hospital to home) | To explore FCs’ experiences with caring for CMC who are initiated on medical technology during a hospital admission. We sought to understand their experiences undergoing the training programme in hospital as well as throughout the process of being discharged and transitioning to new daily life at home. | Semi-structured interviews | Mixed conditions | < 6 years, n = 8 (57.1%) 6 - 12 years, n = 2 (14.3%) 13 - 18 years, n = 4 (28.6%) | 14 interviews  Mothers, n = 11 Fathers, n = 3 | Content analysis |
| Pitch et al., | 2023 | Canada | All health and care services | To gain an improved understanding of the caregiving experience for CMC during the pandemic. We also aimed to provide relevant stakeholders with recommendations from FCs on how to best support their ability to care for their children and family | Semi-structured interviews | Not reported | < 6 years, n = 1 (8.3%) 6 - 12 years, n = 7 (58.4%) 13 - 18 years, n = 4 (33.3%) | 12 participants  Female, n = 11  Male, n = 1 | Four-step content  analysis |
| Scheer et al., | 2024 | USA | All health and care services | To characterize the underrecognized experiences of primary caregivers of CMC in engaging with other members of the child’s caregiving network, thereby informing the design of health IT for the caregiving network. | Semi-structured interviews | Mixed conditions | Not reported | Not reported | Braun and Clarke's  protocol for thematic analysis, secondary analysis |
| Shouldice et al., | 2024 | Canada | An online patient platform | To understand the parental caregiver experience of using Connecting2gether (C2), a web and mobile-based patient-facing platform for parental caregivers of CMC. The objectives were to (1) identify barriers and challenges to platform uptake, (2) understand facilitators of parental use, and (3) evaluate the role of a live platform coach. | Questionnaires | Not reported | Not reported | Parents, n = 37  Mother, n = 30 (81%) Father, n = 7 (19%) | 6 stages for thematic  analysis proposed by Braun and Clarke |
| Sonsteng-Person et al., | 2023 | USA | All health and care services | (1) To record and reflect mothers' identified main concerns and strengths within their lives by taking pictures, (2) To engage mothers in creating and sharing their own knowledge about the conditions of their lives through group discussion and analysis of pictures, (3) To identify and create policy and practice recommendations for hospitals and social care systems working with children with medical complexity. | Photovoice | Mixed conditions | Not reported.  Inclusion criteria states children must be 0 - 6 years old. | Mothers, n = 6 | Qualitative analysis |
| Tager et al., | 2023 | USA | All health and care services | To describe the impact of the COVID-19 pandemic on families of children with medical complexity. | Semi-structured interviews | Not reported | Mean = 8.2 years SD = 3.8 years Range = 2 - 19 years | Mothers, n = 19 Fathers, n = 1 | Interpretive  phenomenological analysis |
| Toly et al., | 2019 | USA | Hospital discharge | To explore how mothers perceive their transition experiences just prior to and during the first three months after initial NICU discharge. | Qualitative, descriptive, longitudinal design | Mixed conditions | Gestational age: Mean = 29.78 weeks SD = 6.43 weeks | Mothers, n = 19 | Descriptive content analysis |
| Van de Riet et al., | 2023 | Netherlands | Hospital discharge | To gain a deeper understanding of the needs and experiences of Dutch CMC parents on hospital-to-home (H2H) transition and integrate these insights with the literature review into an evidence-based H2H care pathway for CMC and their families | Semi-structured, open-ended interviews, descriptive  phenomenological approach | Mixed conditions | Range = 6 months - 10 years | 14 interviews  Mothers, n = 14 Fathers, n = 7 | Thematic analysis |
| Vergo Houlihan et al., | 2024 | USA | All health and care services | No aim specified: The study shares themes from focus groups, in which families described the need for systems to counteract widespread misconceptions and bias they continuously experience in order to achieve meaningful system change | Family-designed,  family-led focus groups | Not reported | Not reported | 127 across 10 groups | Inductive content  analysis |
| Wright-Sexton | 2020 | USA | Paediatric Intensive Care | To better describe the experiences of parents and providers of children with chronic critical illness specifically around isolation during PICU admission | Surveys and semi-structured interviews | Mixed conditions | Mean = 11 years IQR = 4 - 18 years | PICU physicians, n = 7 Nurse practitioners, n = 8 Parents, n = 12 | Constant comparative  analysis |
| Yamoah et al., | 2023 | USA | All health and care services (focus mostly on respite, home care and mental health services) | To explore the role of social support in mitigating parental burnout in mothers of CMC. It also aims to offer guidance to community-based organizations, practitioners, and mental health advocates on how to best allocate resources to provide social support opportunities for mothers of CMC. | Interviews | Mixed conditions | 9 years, n = 1 11 years, n = 1 12 years, n = 1 15 years, n = 1 16 years, n = 1 | Participants, n = 5 | Inductive analysis process, qualitative data analysis, cross case analysis |
