# Supplementary material for: Family Caregivers' Experiences of Services for Children With Medical Complexity: A Systematic Review and Qualitative Evidence Synthesis
Source: Health Expect. 2025 Sep 29;28(5):e70452. doi: 10.1111/hex.70452 (PMC12480434; doi:10.1111/hex.70452)
Supplement: Supplementary file 3 — CASP Quality Checklist. [file HEX-28-e70452-s001.docx]

**Supplementary File 3**

**CASP Quality Checklist**

| **Author and year** | **Clear statement of aims** | **Appropriate qualitative methodology** | **Appropriate design to meet research aims** | **Recruitment strategy  appropriate to research aims** | **Data collected in a way that addressed the research issue** | **Considered relationship  between researcher and participants** | **Considered ethical issues** | **Sufficiently rigorous data  analysis** | **Clear statement of findings** | **Research value** | **Summary** |
| --- | --- | --- | --- | --- | --- | --- | --- | --- | --- | --- | --- |
| **Amar-Dolan et al., 2020** | Yes | Yes | Yes | Yes. *Only one method of recruiting.* | Yes | Yes. *States physician researcher had no longitudinal clinical relationship, which may have caused some increased bias in the results.* | Yes | Yes. *Explained clearly the steps used for the analysis.* | Yes | States a clear proposal for improvements. Clearly identifies what parents struggle with the most. Suggests some good ways on how to overcome this, such as simulations for training staff and families. | **No concerns** |
| **Ames et al., 2023** | Yes | Yes | Yes | Yes | Yes. *Data was collected by two interviewers, could have variation in data collected.* | Can't tell. *No mention of the backgrounds of interviewers.* | Yes | Yes. *Transcripts were dually coded,  clearly explains steps used for the data analysis.* | Yes | Highlights medical professionals do not have enough training and therefore are hesitant to treat children with medical complexity. It also shows that clinicians make assumptions about the child's diagnosis rather than individually getting to know the child. This study shows that more education is needed to combat disability-based discrimination. | **No major concerns** |
| **Ames et al., 2024** | Yes | Yes | Yes | Yes. *Multiple methods used to recruit participants.* | Yes. *Data was collected by two interviewers, could have variation in data collected.* | No. *Mentions both interviewers  were paediatric critical care physicians, does not mention if they have been involved with child's care. Could mean participants are not as inclined to explain all discriminations due to power imbalance.* | Yes | Yes | Yes | Research is valuable as clearly states what should happen in the future to minimise discrimination, such as educating clinicians about the discrimination that families experience and creating standards of accountability in healthcare systems. It proposes policymakers must continue to address the discriminations. | **No major concerns** |
| **Boss et al., 2020** | Yes | Yes | Yes | Yes. *Only one method of recruiting.* | Yes | No. *States the initials of person who conducted interviews, doesn’t state their occupation or relation to participant.* | Yes | Yes | Can't tell. *Speaks about results from  interviews in discussion, however there is no mention of the gaps in the conclusion, which was a significant* *part of the discussion.* | Highlights to clinicians the importance of family centred care and the need to emphasise to families that they are involved in the care plan. Families should be encouraged to have conversations with staff about their preferences while caring for their child. It shows the benefits of PHHC and its potential while stating the gaps in the service, showing it is a service that is worth improving. | **No major concerns** |
| **Buchanan et al., 2022** | Yes | Yes | Yes | Yes. *Only one method of recruiting. Did not mention why the specific conditions were chosen for the inclusion criteria.* | Yes | No. *States the initials of person who conducted interviews, doesn’t state their occupation or relation to participant.* | Yes | Yes | Yes | Clearly states how this research is useful and suggests ways to improve day to day interactions between healthcare professionals and caregivers. It suggests that the caregivers' experiences should be considered more when deciding on a care plan. | **No major concerns** |
| **Cady et al., 2017** | Yes | Yes | Yes. *Specifically stated why focus  groups were chosen over individual interviews.* | Yes | Yes | Can't tell. *States focus groups were  conducted by an experienced interviewer, doesn't state if they are a clinician or if they have a relationship with participant.* | Yes | Yes. *States how disagreements were reviewed.* | Yes | Conclusion identifies the ideal situation where there is a platform for sharing comprehensive health data system-wide and states this is not achievable currently. Suggests further work could go into this. However, in the meantime it would take a large responsibility off of the parents if clinicians were able to effectively communicate the patient's needs with each other. This would help alleviate some of the stress caregivers experience. | **No major concerns** |
| **Currie et al., 2023** | Yes | Yes | Yes | Somewhat. *Used maximum variation to try  and represent as many perspectives as possible. Does not state why some people did not agree to being interviewed. Does not state how participants were approached.* | Can’t tell. *States modifications were  made but does not explain what was changed and why this was done.* | No. *Does not mention who conducted interviews.* | Yes | Yes | Yes. *States the disruption of care  coordination services had negative impacts on families. The study mentions children with neurodevelopmental disabilities were disproportionally impacted but the study does not explain why this was the case.* | The study shows how beneficial care coordination is to families. The paper shows that there is a need for care coordinators to be considered as an essential service. | **No major concerns** |
| **Dewan et al., 2023** | Somewhat - Vague aim 'to describe  experiences' | Yes | Yes | Yes. *Explained how they targeted  variability. Doesn't state why the two parents did not sign the consent form or complete the interview.* | Yes | No. *No mention of who conducted  the interviews* | Yes | Yes | Yes | States this is the first paper that discusses pediatric medical traumatic stress. Has quotes from participants stating what they would like to see improved. This is very useful to ensure families feel supported. | **No major concerns** |
| **Fong et al., 2023** | Yes | Yes | Yes. *Explains the benefits of using  interpretive description.* | Yes | Yes | Can't tell. *Mentions the first and second author conducted the interviews but does not state their background or relationship with participant.* | Yes | Yes | Yes | This paper shows that although there are benefits to virtual care such as being more convenient and less of a financial burden, some aspects of care are viewed as less effective virtually, such as speech and language therapy. | **No major concerns** |
| **Fong et al., 2024** | Yes | Yes | Yes | Can’t tell. *States participants were recruited in several ways but only mentions online advertising.* | Yes | No. *States initials of who conducted the interviews but not their background or relationship with participant.* | Yes | Yes | Yes | This paper highlighted the importance of training healthcare professionals to be able to use virtual care effectively. It also clearly points out the areas that need to be improved as well as the need for continuous reflection and evaluation on how virtual care is delivered to patients. | **No major concerns** |
| **Foster et al., 2022** | Yes. T*here are two aims of the paper.* | Yes | Yes | Yes. *Two methods of recruiting used.* | Can’t tell. *Modifications were made but  does not explain what changes were made.* | Can't tell. *States interviews were conducted by study staff but doesn't mention their background.* | Yes | Yes | Yes | Table 3 clearly shows where improvements need to be made. The study suggests the need for further research focusing on parental wellbeing and support structures. It highlights to clinicians that there are flaws in the systems that are supposed to support families, therefore meaning there is a need to review these current systems that have been put in place. | **No major concerns** |
| **Frush et al., 2023** | Yes. *There are two aims of the paper.* | Yes | Yes | Can’t tell. *Does not mention how participants were recruited, just states they were participants in a pilot study of posthospitalisation TMVV.* | Yes. *They justified using Colaizzi’s  Descriptive Phenomenological Method.* | No. *Does not mention who conducted interviews.* | Yes | Yes | Yes | This paper highlights the benefits of telemedicine virtual visits as it promotes independence of the caregivers as they feel they gain back a sense of control over their schedule. | **No major concerns** |
| **Golden et al., 2012** | Yes | Yes | Yes | Yes | Yes | No. *Does not mention who conducted interviews.* | Yes | Yes | Yes | Paper highlights that care coordination is a major stress factor for families, and clinicians could alleviate this burden by communicating more efficiently with the families and between teams. Paper emphasised that communication is a major skill needed for working with CMCs and something that caregivers greatly appreciate. | **No major concerns** |
| **Hagvall et al., 2016** | Yes | Yes | Yes | Yes. *Does not say why parents did  not participate.* | Yes. *Gave examples of some of the  questions used. Open ended questions.* | Yes | Yes | Yes. *Explains how themes were  derived.* | Yes | Paper very clearly states ways to improve and suggests a nursing model. Highlights need for supporting the families. | **No concerns** |
| **Hlyva et al., 2021** | Yes | Yes | Yes | Can’t tell. *Does not mention how  participants were recruited.* | Yes | Can't tell. *Interviews were conducted by  an experienced qualitative.* *researcher, assuming they do not have a relationship with participants.* | Yes | Yes | Yes | Paper highlights the families' appreciation for care coordinators. Research is very valuable as it states that the care coordination team is something that is highly valued by families. | **No major concerns** |
| **Hobson et al., 2011** | Yes. *There are three aims of the  paper.* | Yes | Yes | Yes. *Only one method of recruiting.* | Yes | Yes. *Mentions interviewers do not know participants.* | Yes | Yes | Yes | Paper is very valuable as it is about fathers' perspectives which is underrepresented. States that they would appreciate more support around intimate personal care for their child. | **No concerns** |
| **Keilty et al., 2018** | Yes | Yes | Yes | Can’t tell. *Does not mention how  participants were recruited. Does not mention why not all of the eligible parents participated.* | Yes | Can't tell. *Conducted by a researcher but  does not state if they are previously known to participants.* | Yes | Yes | Somewhat. *There is a summary paragraph  at the end but it is not under a conclusion heading, it is included in the discussion.* | Paper mentions the benefits of using unregulated caregivers as they have more flexible hours and families feel as though they are more willing to learn from parents and adhere to their wishes. | **No major concerns** |
| **Leary et al., 2020** | Yes | Yes | Yes | Somewhat*. Is not clear how participants were recruited. I believe nurses and researchers approached families while they were in the hospital, but this isn't clearly stated.* | Yes. *Gave examples of the  modifications.* | Yes. *Clearly states none of the interviewers were involved in the medical teams.* | Yes | Yes. *In-depth description of the  analysis process.* | Yes | This paper mentions the challenges with transitioning the care form the hospital to the home and the difficulties of discontinuity and poor communication between healthcare services. | **No major concerns** |
| **McLorie 2023** | Yes | Yes | Yes | Yes | Yes | Yes. *Conducted by a researcher not previously known to parents. Eliminates fear parents may have of speaking up to healthcare team.* | Yes | Yes | Yes | Research is very valuable. Clearly states the specific areas where parents feel unsupported, and highlights need for healthcare professionals to consider psychological support and informing carers where to go for information. | **No concerns** |
| **Mendes et al., 2013** | Yes | Yes | Yes | Can’t tell. *Does not mention how  participants were recruited.* | Yes | No. *Does not mention who  conducted interviews.* | Yes | Yes | No. *There is no final conclusion  paragraph in this paper.* | Paper highlights the importance of families being able to trust their nurses by allowing the families to feel included in the care of their child and for their experience to be acknowledged. Parents in the paper suggest that nurses should be able to 'fit in' to their household routines. | **No major concerns** |
| **Mitchell et al., 2022** | Yes. *There are three aims of the  paper.* | Yes | Yes | Yes. *Multiple methods used to recruit participants.* | Yes. *Explained the use of auto-driven photo-elicitation interviews.* | No. *Does not mention who  conducted interviews.* | Yes | Yes | Yes |  | **No major concerns** |
| **Moyes et al., 2022** | Yes | Yes | Yes | Yes | Yes | Somewhat. *Interviews were conducted by the first author, does not mention if they have a relationship.* | Yes | Yes | Yes | Paper shows that parents feel more confident in clinicians if they feel as though they are guiding them through their own expertise and experiences of caring for their own child. Shows that communication between parents and healthcare teams needs to be improved so parents feel heard. | **No major concerns** |
| **Nageswaran et al., 2022** | Yes | Yes | Can’t tell. *States the methodology was  described on a previous paper. May have been clearer to explain it on this paper too.* | Can't tell | Yes | No. *Does not mention who  conducted interviews and if there was a relationship between interviewer and participant.* | Yes | Yes | Yes. *Very short introduction, could  have given some examples of the specific challenges in communication.* | This paper shows that translators are not used/available  consistently. Paper shows that there are many boundaries when communicating with healthcare professionals, pharmacies and services. | **No major concerns** |
| **Page et al., 2020** | Yes | Yes | Yes | Can't tell | Yes | No. *Does not mention who  conducted interviews.* | Yes | Yes | Yes | Paper highlights that caregivers believe they have to advocate for their child and 'fight the system'. It also mentions the lack of sleep that caregivers get and points out the dangers associated with this. | **No major concerns** |
| **Rennick et al., 2019** | Yes | Yes | Yes. *Justified why they chose to use  interpretive description.* | Yes | Yes | Yes. *Mentions interviewer did not  work in the PICU.* | Yes | Yes | Yes | Paper shows the importance of communication between  healthcare teams and parents. Parents described feeling more comfortable when they were known by the staff and when they would consider their opinions on decision for care of their child. It also briefly mentions the psychological challenges that parents are faced with. | **No concerns** |
| **Sherman et al., 2024** | Yes. *There are three aims of the  paper.* | Yes | Yes | Yes | Yes | Can't tell. *The first author conducted the  interviews, does not mention if the participant is previously known to interviewer. States interviewer has a background of working in children's hospitals.* | Yes | Yes | Yes | Paper shows that parents would appreciate support groups to be offered as they found peer support that they found online to be useful. Paper also highlights that caregivers do not feel as though the trach-care training they received reflected the reality of their situation. | **No major concerns** |
| **Thomas et al., 2012** | Yes | Yes | Yes | Can't tell | Can’t tell. *Explain that interviews were recorded and transcribed. No mention of use of topic guide and no discussion of data saturation.* | Can't tell. *Mentions that interviewer was a lecturer but minimal discussion of the role of the researcher.* | Yes | Can't tell. *Very limited description of analysis process. Results section is clear with use of quotes to illustrate the themes.* | Yes | Paper shows the value of respite care for families and identifies inconsistencies in the amount and type of respite provision offered to families. | **Some concerns** |
| **Welsh et al., 2014** | Yes | Yes | Yes | Yes | Yes | No. *Does not mention who  conducted interviews and if they had a relationship with participants.* | Yes | Yes | Yes | Paper shows the importance of respite for careers and their other children, but also shows there are some challenges associated with receiving this respite including the different rules and regulations for each service provider and the need for specialised equipment in the families' home. | **No major concerns** |
| **Yu et al., 2022** | Yes | Yes | Yes | No | Yes | Can't tell. *Mentions interviewer has training in paediatrics, palliative care and clinical research, does not mention if they have a relationship with families.* | Yes | Yes | Somewhat. *There is a summary paragraph  at the end but it is not under a conclusion heading, it is included in the discussion.* | This paper suggests that care coordinators can act as a voice for parents when communicating with clinicians as the healthcare team were more likely to follow a care plan even id it conflicts with a subspecialty when it is explained by the care coordinator rather than the parents. This shows it is important that caregivers do have someone to advocate for them and their child. | **No major concerns** |
